# Clotting Promotes Glioma Growth and Infiltration Through Activation of Focal Adhesion Kinase

**DOI:** 10.1158/2767-9764.CRC-24-0164

**Published:** 2024-12-13

**Authors:** Lynn M. Knowles, Carolin Wolter, Stefan Linsler, Simon Müller, Steffi Urbschat, Ralf Ketter, Andreas Müller, Xiangda Zhou, Bin Qu, Sebastian Senger, Jürgen Geisel, Tim Schmidt, Hermann Eichler, Jan Pilch

**Affiliations:** 1 Institute of Clinical Hemostaseology and Transfusion Medicine, Saarland University and University Medical Center, Homburg, Germany.; 2 Department of Neurosurgery, Saarland University and University Medical Center, Homburg, Germany.; 3 Clinic for Diagnostic and Interventional Radiology, Saarland University and University Medical Center, Homburg, Germany.; 4 Biophysics, Center for Integrative Physiology and Molecular Medicine (CIPMM), Saarland University and University Medical Center, Homburg, Germany.; 5 Clinical Chemistry and Laboratory Medicine, Saarland University and University Medical Center, Homburg, Germany.

## Abstract

The tumor architecture of high-grade gliomas is shaped by tumor cell necrosis, invasive growth, and the leakage of a fibrin-rich edema from poorly organized tumor blood vessels. In this study, we demonstrate a marked upregulation of clot formation in the interstitial spaces of tumor tissues from patients with glioblastoma (GBM) whereas a tumor-free brain is essentially devoid of fibrin. The accumulation of fibrin in tumor interstitial spaces is functionally relevant as we demonstrate increased infiltration and growth of primary GBM cells after embedding in a 3-dimensional matrix made of fibrin *ex vivo*. Additionally, we detected accelerated tumor growth after implanting GBM cells together with clotted plasma in brains of immunodeficient mice whereas GBM development in clotting-deficient hemophilia mice was delayed. GBM growth correlated with the outgrowth of invadopodia and their adhesive interactions with the 3-dimensional clot matrix, which was mediated by integrins β1 and β3 and their common downstream target focal adhesion kinase (FAK). Knocking down FAK with CRISPR Cas9 caused an upregulation of p21/p27 cell-cycle inhibitors, strong growth inhibition in cultured GBM cells, and sustained antitumorigenic effects in orthotopic GBM xenografts *in vivo*. These results go hand in hand with genomic data from The Cancer Genome Atlas that indicate increased clotting activity and reduced patient survival in glioma subgroups with high integrin β1 and β3 expression. We therefore conclude that clotting in glioma interstitial spaces provides tumor cells with a potent proliferative stimulus that can be reversed by targeting the adhesive machinery of GBM cells via inhibition of FAK.

**Significance:**

High-grade gliomas are associated with intratumoral thrombosis, tumor cell necrosis, and hemorrhage. The resulting blood clot serves as an adhesive matrix for glioma cell integrins that activate FAK. Knocking down FAK with CRISPR cas9, on the other hand, is highly effective at halting GBM growth in mice.

## Introduction

High-grade gliomas [World Health Organization (WHO) grade 3 or 4] are malignant brain tumors that are derived from glial progenitors, oligodendrocytes, or astrocytes (1). The most frequent glioma in adults is glioblastoma (GBM), which accounts for 60% to 70% of newly diagnosed brain tumors, followed by anaplastic astrocytoma with 10% to 15% and anaplastic oligodendroglioma or oligoastrocytoma with together 10% of the cases. The prognosis of malignant glioma is poor with an overall survival (OS) of 12 to 15 months for GBM and 2 to 5 years for anaplastic glioma. A hallmark of glioma is the diffuse infiltration of the neuropil, which regularly prevents complete surgical removal even in premalignant lesions (2). Although WHO grade 2 isocitrate dehydrogenase (IDH)-mutant glioma can be treated with vorasidenib, low-grade astrocytomas (WHO grade 2) progress over time into anaplastic astrocytomas (WHO grade 3) and secondary GBMs (WHO grade 4), which are characterized by vastly increased proliferation and intracerebral dissemination (3). Gliomas can invade as individual cells or in small groups using a mechanism that is reminiscent of metastatic tumor cells that undergo epithelial to mesenchymal transition (EMT; 4). Although gliomas are generally considered nonmetastatic, they are able to activate transcriptional programs known to promote mesenchymal cell functions that are associated with increased tumor cell invasion, a high rate of proliferation, and poor patient survival (5). Taken together, these data indicate that tumor cell invasion is a major aspect of glioma pathogenicity and that defining the underlying adhesive mechanisms could lead the way to reversing the diffuse growth pattern of this malignancy.

Among the most prominently upregulated proinvasive factors in glioma is CD44, which takes place early during gliomagenesis and promotes glioma invasion into the neuropil through interaction with its ligand hyaluronan in the extracellular matrix (ECM) of the brain (6). However, interactions with hyaluronan are not sufficient to mediate glioma infiltration alongside neuronal, astrocyte, or white matter tracks without cooperation with integrin cell adhesion receptors, which provide directed traction after binding to fibronectin, vitronectin, or other fibrillar ECM proteins overexpressed in gliomas (7). In agreement, it has been shown that integrins that interact with these ECM proteins are upregulated in glioma and that their overexpression correlates with decreased survival of patients afflicted with glioma (8, 9). Laminin-binding integrins, on the other hand, are predestined to mediate adhesive interactions with the basement membrane in perivascular spaces, which represent another important cudgel for glioma infiltration of the brain parenchyma (10). As the perivascular spaces of the brain provide specialized docking places for glioma stem cells (GSC), it is not surprising that GSCs overexpress integrins α6 and α7, which in turn activate intracellular signaling cascades through adhesive interactions with laminin in order to promote glioma growth and invasion (11, 12). Other functionally relevant integrins on GSCs include integrins αvβ3, αvβ5, αvβ8, and α2β1, suggesting that targeting adhesive interaction of glioma cells with their respective ECM could have a significant impact on controlling infiltration and growth of glial brain tumor cells (8, 13–15).

The basic components of the brain ECM are hyaluronan, chondroitin sulfate proteoglycans, and tenascin-R, which together form a 3-dimensional (3D) scaffold that promotes neurite outgrowth and prevents infiltration of inflammatory as well as tumor cells (16). The latter process requires adhesive interactions of integrins with fibrous glycoproteins that are not present in the normal brain but become upregulated during tumorigenesis, inflammation, and injury (17–19). As such, remodeling of the brain ECM is contingent on the secretion of polymeric glycoproteins such as tenascin-C, fibronectin, collagen, and laminin by tumor, stromal, and endothelial cells (10, 20–22). A second mode of brain tissue remodeling results from circulating adhesion proteins such as fibrinogen, plasma fibronectin, and vitronectin, which extravasate together with coagulation factors, thereby forming a provisional fibrin matrix that promotes the survival of tumor and inflammatory cells (18). Blood clotting is also common in gliomas that upregulate tissue factor and plasminogen activator inhibitor due to oncogene activation (23, 24). These procoagulant and antifibrinolytic proteins promote thrombotic occlusion of tumor blood vessels and subsequent extravascular clotting in tissue voids generated by ischemic tumor cell necrosis (24, 25). The underlying state of hypoxia in turn represents a potent stimulus for tumor angiogenesis, with the resulting microvascular hyperplasia feeding directly into the vicious cycle of plasma leakage, ischemia, and interstitial clotting (26, 27). This process seems to be clinically relevant as there is a strong correlation between the survival of patients with glioma and the extent of necrosis as well as clotting in the corresponding tumor tissues (28, 29).

We previously demonstrated that blood clotting specifically supports lung metastasis whereby fibrin generates a provisional ECM that promotes tumor cell invasion and proliferation (30, 31). The permissive function of blood clotting depended on a constitutively active form of integrin αvβ3 that cooperates with fibronectin in tumor cells surrounded by fibrin. Integrin αvβ3 and fibronectin, in turn, have been shown to be involved in glioma progression (8, 20). Based on these data, we hypothesized that fibrin can serve as a conduit for malignant brain tumor cells by providing a permissive ECM for GBM adhesion, invasion, and proliferation. In this context, we identified a relevant function of focal adhesion kinase (FAK) downstream of glioma integrins β1 and β3 for glioma growth.

## Materials and Methods

### IHC

Anatomically certified paraffin tissue sections from patients with astrocytoma grade 2, astrocytoma grade 3, GBM, or adjacent healthy brain (three patients each; Supplementary Table S1) were purchased from Pantomics, Inc. Slides (4 μm) were baked at 60°C for 30 minutes, deparaffinized using 100% xylene, and rehydrated in isopropanol washes serially diluted from 100% to 70% and rinsed first in H_2_O and then in PBS containing 0.005% Tween 20. Antigen retrieval was performed by incubating the tissue sections with citrate buffer pH 6.0 for 30 minutes at 95°C. The slides were cooled at room temperature, washed in PBS containing 0.005% Tween 20, and air-dried. Tissues were permeabilized with 0.5% Triton X-100 for 10 minutes, blocked with 2% BSA for 20 minutes, and stained with an antifibrinogen (Nordic-Mubio, catalog # GAHu/Fbg/7S) or isotype control (Thermo Fisher Scientific, catalog # 31245, RRID: AB_10959406) for 1 hour followed by incubation with Alexa Fluor 488–conjugated secondary antibody (Thermo Fisher Scientific, catalog # A21222, RRID: AB_10373853). Nuclei were stained with mounting media containing 4′,6-diamidino-2-phenylindole [DAPI (Sigma-Aldrich, RRID: SCR_008988, catalog # F6057-20 ML)]. Fluorescence was visualized on a Nikon Eclipse Ni fluorescent microscope equipped with NIS Elements software (Nikon, RRID: SCR_014329), and percent area was calculated using Image J software (RRID: SCR_003070).

### Cell culture

U87MG, U373MG, and U343MG GBM cells were maintained in minimal essential medium (catalog # 31095052) supplemented with 10% FBS (catalog # 10500064), L-glutamine (catalog # 25030024), minimal essential medium vitamins (catalog # 11120037), nonessential amino acids (catalog # 11140035), sodium pyruvate (catalog # 11360070), and antibiotics (catalog # 15140122) from Gibco (Thermo Fisher Scientific, RRID: SCR_008452). Cell line authenticity was verified by short tandem repeat genotyping using Promega PowerPlex 21 System [Eurofins Genomics; U87MG (ATCC, catalog # HTB-14), U373MG (ATCC, catalog # HTB-17); and U343MG (DSMZ, catalog # ACC-408)]. GL-261 mouse glioma cells (DSMZ–German Collection of Microorganisms and Cell Cultures GmbH, catalog # ACC-802, RRID:CVCL_Y003) were maintained in DMEM media (Thermo Fisher Scientific, catalog # 41966029) containing 10% FBS and antibiotics. All cell lines were confirmed to be *Mycoplasma*-free using Lonza MycoAlert Detection kit (Lonza, RRID: SCR_000377, catalog # LT07-418). Cells were cultured at 37°C under a humidified, 5% CO_2_ atmosphere.

### Primary tumor cells

Patients diagnosed with glioma were operated on at the Department of Neurosurgery at the Saarland University from November 2014 to October 2016. Following resection, tumors were inspected by a neuropathologist and classified according to the 2007 WHO classification of tumors of the central nervous system (Table 1; ref. 32). Tumor samples from patients with astrocytoma grade 2, astrocytoma grade 3, and GBM were minced using a scalpel, and the subsequent cell suspension was cultured in DMEM containing 10% FBS, nonessential amino acids, and antibiotics. Study approval was received from the local Ethics Committee (Kenn-Nr. 93/16, Ethik-Kommission Ärztekammer Saarland) and conducted in accordance with the Declaration of Helsinki ethical guidelines. Written informed consent was received by all participants.

**Table 1:** Clinicopathologic data of patients with glioma

Patient	Age	Sex	Cell type	WHO grade	MGMT	IDH1	p53
1	46	M	Astrocytoma	Grade 2	—	Mut	Pos
2	37	M	Astrocytoma	Grade 2	—	WT	Pos
3^a^	69	M	GBM	Grade 4	—	WT	Neg
4	76	M	Astrocytoma	Grade 3	—	WT	Pos
5	33	F	Astrocytoma	Grade 3	—	Mut	Pos
6	61	M	Astrocytoma	Grade 3	—	WT	Pos
7	72	F	GBM	Grade 4	Met	WT	Pos
8	54	M	GBM	Grade 4	—	WT	Neg
9	84	F	GBM	Grade 4	Met	WT	Pos
10	53	F	GBM	Grade 4	—	—	—
11	63	M	GBM	Grade 4	—	WT	Neg
12	48	M	GBM	Grade 4	—	Mut	Pos
13	56	M	GBM	Grade 4	—	WT	Pos
14	59	M	GBM	Grade 4	—	WT	Pos
15	79	M	GBM	Grade 4	—	WT	Neg
16	74	M	GBM	Grade 4	—	WT	—
17	71	F	GBM	Grade 4	—	WT	Pos

Abbreviations: F, female; M, male; Met, methylated; MGMT, O6-methylguanine-DNA methyltransferase; Mut, mutant; Neg, negative; Pos, positive; p53, tumor protein p53; WT, wild-type; — , unknown.

a Originally classified as an astrocytoma grade 2 but reclassified as GBM grade 4.

### 3D cell culture

Fibrin gels were generated by suspending 2 × 10^4^ tumor cells in 3.8% citrated human blood plasma (“plasma clot”; United States Biological, RRID: SCR_013653, catalog # P4252-30A) or a solution of 2 mg/mL human fibrinogen (“fibrin clot”; Enzyme Research Laboratories, catalog # FIB 3). A mixture of 2.5 U/mL thrombin (Sigma-Aldrich, catalog # T4393) and 2 to 3 mmol/L CaCl_2_ was added to each suspension to induce clotting and the nascent plasma, and fibrin clots were transferred in 15 μL portions onto nontissue culture–treated plates before clotting was complete. For studies using Matrigel basement membrane matrix (Corning, catalog # 354234), cells were mixed with ice-cold Matrigel and plates were inverted and incubated at 37°C for 25 minutes for the basement membrane gels to solidify. All embedded tumor cells were covered with media, and five clots per treatment were monitored daily at three designated areas by phase-contrast microscopy using a Zeiss PrimoVert microscope with an attached camera and Zen light 2012 software (RRID: SCR_013672). Completely spread tumor cells were classified as invadopodia-positive based on their elongated and stellated shape whereas round tumor cells with or without rudimentary invadopodia were classified as invadopodia-negative. The results from this analysis were depicted as the ratio of completely spread, invadopodia-positive tumor cells to the total number of tumor cells per microscopy field and denoted as invadopodia (%). Cell growth was monitored by counting the total number of embedded tumor cells per microscopy field and depicted as fold increase above cell numbers at baseline (proliferation × 0 hour). Where indicated, fibrin clots were incubated in the presence of 3 mmol/L of the plasmin inhibitor tranexamic acid (TXA; Carinopharm, catalog # 4150165338107) or 10 μmol/L of the matrix metalloproteinase (MMP) inhibitor GM6001 (Merck Millipore, catalog # CC1010). Live-cell imaging was performed over 3 days using ImageXpress Micro XLS Widefield High-Content Screening System (Molecular Devices) to measure invadopodia formation and proliferation over time. Phase-contrast images from four optic fields were taken every 10 minutes, and videos were generated using Image J 1.53k software. In addition, the 10-minutes frames were uploaded into Imaris software 9.0 (Oxford Instruments, RRID: SCR_007370). Invadopodia formation was established using Imaris software by tracking 10 to 20 individual cells within the first 16 hours after embedding in plasma clot, fibrin clot, or Matrigel. This allowed us to count the cumulative number of invadopodia generated per cell over an average of 4 to 6 hours. In addition, we measured the largest diameter of GBM cells as an approximate measure of invadopodia length 16 hours after embedding in plasma clot, fibrin clot, or Matrigel. Cell length was depicted per cell as average from the number of cells per optical field using ImageJ. Proliferation was assessed using Imaris software by tracking the change of cell number over 3 days after embedding.

### MTS (**3-(4,5-dimethylthiazol-2-yl)-5-(3-carboxymethoxyphenyl)-2-(4-sulfophenyl)-2H-tetrazolium) **assay

Cells (1 × 10^3^) were plated in 96-well plates, and proliferation was measured after 4 days using CellTiter 96 AQueous One Solution Cell Proliferation Assay kit (Promega, catalog # G3580) according to the manufacturer’s directions.

### siRNA-mediated gene silencing

Tumor cells were transfected with 25 nmol/L siRNA against β1 integrin (Dharmacon, ON-TARGETplus SMARTpool, # L-004506-00-0005), β3 integrin (Dharmacon, ON-TARGETplus SMARTpool, # L-004124-00), *PTK2*, the gene for FAK (Dharmacon, ON-TARGETplus SMARTpool, # L-003164-00), or a nontargeting control (Dharmacon, ON-TARGETplus, # D-001810-10-20). siRNA was diluted in Opti-MEM reduced-serum medium (Thermo Fisher Scientific, catalog # 31985047) and transfected in the presence of lipofectamine 2000 (Thermo Fisher Scientific, catalog # 11668027) for 5 hours. Cells were then washed and grown for an additional 67 hours under normal culturing conditions using minimum essential medium complete media. siRNA-transfected cells were embedded in plasma clot and fibrin clot as described above and probed for invasion and cell proliferation. Knockdown efficiency was determined by Western blot analysis.

### Generation of stable FAK knockout cells by CRISPR-cas9

Tumor cells were transfected with an all-in-one plasmid from ATUM (catalog # Hs:8:141,745,351–141,745,389) containing the pD1431-APuro vector (pD1431-APuro: EF1a-Cas9N-2A-Puro, Cas9-ElecD) and two single-guide RNAs (5′-TAA​ACC​TGG​GCC​GCC​TGC​TG-3′ and 5′-ACT​TAA​AGC​TCA​GCT​CAG​GT-3′) targeting FAK. Briefly, cells were seeded in antibiotic-free minimum essential medium media and grown to 70% to 90% confluency prior to transfection with a 1.5:1 ratio of lipofectamine 3000 (Thermo Fisher Scientific, catalog # L3000008) to DNA according to the manufacturer’s recommendations. After 24 hours, the transfection media was removed, and cells were allowed to recover for an additional 24 hours before being placed under selection with 1 μg/mL puromycin (Thermo Fisher Scientific, catalog # A1113803) for 7 days. The surviving cells were allowed to grow for 2 to 4 weeks before single-cell clones were generated in 96-well plates using serial dilution. These single clones were expanded, and FAK knockdown was verified by Western blot analysis. Three FAK-negative clones were randomly selected for subsequent *in vitro* and *in vivo* experiments.

### Western blot analysis

Cells were lysed using 2× SDS sample lysis buffer (125 mmol/L Tris, 10% glycerol, 4% SDS, and 1.8% 2-mercaptoethanol) and harvested by scraping. Proteins were separated by SDS-PAGE, transferred to a PVDF membrane and stained with 0.1% Ponceau S solution (Sigma-Aldrich, catalog # 6226-79-5) to ensure equal sample loading. The membrane was blocked with 5% non-fat dry milk or 5% BSA in PBS containing 0.1% Tween 20 for 1 hour at room temperature and probed for anti-integrin β1 (Cell Signaling Technology, catalog # 9699, RRID: AB_11178800), anti-integrin β3 (BD Biosciences, catalog # 611140, RRID: AB_398451), anti-integrin β5 (Cell Signaling Technology, catalog # 3629, RRID: AB_2249358), anti-integrin αV (BD Biosciences, catalog # 611012, RRID: AB_398325), anti-FAK (Cell Signaling Technology, catalog # 3285, RRID: AB_2269034), anti-pFAK Y397 (Thermo Fisher Scientific, catalog # 700255, RRID: AB_2532307), anti-pFAK Y925 (Cell Signaling Technology, catalog # 3284, RRID: AB_10831810), or α-tubulin (Sigma-Aldrich, catalog # T-6199, RRID: AB_477583) overnight at 4°C. Immunoreactivity was detected using HRP conjugated anti-mouse (Bio-Rad, catalog # 1706516, RRID: AB_2921252) or anti-rabbit (Jackson ImmunoResearch Labs, catalog # 111-035-003, RRID: AB_2313567) secondary antibodies and developed on BioImaging System GeneGenome HR 55000 (Syngene) by enhanced chemiluminescence using Amersham ECL Prime Detection Reagent (Cytiva, catalog # RPN2236).

### Immunocytochemistry

Fibrin-embedded tumor cells were cultured for 2 days and then fixed with ice-cold 4% formaldehyde, permeabilized with 0.5% Triton X-100, and blocked in 3% BSA overnight at 4°C. Cells were stained with anti-cortactin (Thermo Fisher Scientific, catalog # PA5-27134, RRID: AB_2544610) or control IgG (R&D Systems, catalog # AB-105-C, RRID: AB_354266) overnight at 4°C followed by 3 hours of incubation with F(ab′)2 Alexa Fluor 488 (Thermo Fisher Scientific, catalog # A-11070, RRID:AB_2534114) together with Alexa Fluor 546 phalloidin (Invitrogen, catalog # A22283) to stain for F-actin. Nuclei were stained with mounting media containing DAPI. All images were taken with a Zeiss Cell Observer deconvolution microscope equipped with ImageJ 1.53k software and analyzed using Zen 3.2 (Zeiss).

### Clotting *in vitro*

Human citrated plasma (470 μL) was transferred to 5-mL polystyrol tubes and supplemented with or without 3 mmol/L CaCl_2_ and/ or 5 × 10^5^ U87MG tumor cells. After vortexing for 3 seconds, the tubes were incubated for 15 minutes at room temperature and then checked for the presence or absence of clotting. Clotting time was measured by the mechanical ball method by adding U87MG cells (1 × 10^5^–1 × 10^7^ cells) in 100 μL plasma to measuring cuvettes (Diagnostica Stago S.A.S., catalog # 38876) containing a stainless steel ball (Diagnostica Stago S.A.S., catalog # 26441). The clotting reaction was initiated by the addition of 3 mmol/L CaCl_2,_ and cuvettes were gently agitated back and forth until the steel ball ceased movement and the suspension had clotted. Clotting time was recorded using a stop watch.

### Mice

Female athymic Rj:ATHYM-Foxn1nu/nu mice (catalog # SM-ATH-F, RRID: IMSR_RJ:ATHYMIC-NUDE) were purchased from Janvier Labs. Male hemophilia A knockout mice with a severe coagulation factor VIII deficiency were purchased from Jackson Laboratories (B6 129S-F8tm1Kaz/J, catalog # 004424, RRID: IMSR_JAX:004424) along with age-matched control mice (B6 129SF2/J, catalog # 101045, RRID: IMSR_JAX:101045; ref. 33). The mice (6–8 weeks old) were housed in isolated ventilated cages and maintained on a standard pellet diet (Altromin) and water *ad libitum*. All experiments were approved by the local governmental animal welfare committee (Landesamt für Verbraucherschutz, Abteilung C Lebensmittel und Veterinärwesen, Saarbrücken, Germany; Permit Number: 05-2021) and were conducted in accordance with the European legislation on protection of animals (Guide line 2010/63/EU) as well as the NIH Guidelines for the Care and Use of Laboratory Animals (http://oacu.od.nih.gov/regs/index.htm. Eighth Edition; 2011).

### Tumor cell implantation

Athymic Rj:ATHYM-Foxn1nu/nu mice were intracranially injected with U87MG, U373MG, and U343MG cells or the respective CRISPR-Cas9–treated variants. Hemophilia and control mice were injected with GL-261 cells. Mice were anesthetized with 100 mg/kg ketamine (Serumwerk Bernburg AG) and 12 mg/kg xylazine (Wirtschaftsgenossenschaft Deutscher Tierärzte), and a sagittal incision was made to reveal the underlying skull. A small hole was drilled into the scull over the right parietal lobe 2 mm to the right of the sagittal suture and 1 mm behind the coronal suture. Tumor cells (5 × 10^5^) suspended in 5 μL serum-free cell culture media or citrated blood plasma were injected into the parietal lobe using a 10 μL Hamilton syringe that was inserted approximately 2 to 3 mm into the brain tissue. The tumor cell–plasma suspension was supplemented with 3 mmol/L CaCl_2_ immediately before intracranial injection to allow for subsequent clotting *in situ*. Alternatively, tumor cells were suspended in the soluble fraction of clotted plasma that was generated by contact activation of blood plasma *in vitro* and then injected into the brain of athymic nude mice. After completion of the tumor injections, skin wounds were closed with 2 to 3 single-bottom seams, and the mice were monitored until they regained normal activity. During the following weeks, the mice were scored continuously for weight loss, behavioral changes, hunching, and paralysis to assess the tumor burden. Euthanasia was performed when the severity score indicated increasing tumor burden.

### MRI

Mice were examined in a horizontal bore 9.4T MRI scanner for small animals (Biospec 94/20USR, Bruker Biospin) run with ParaVision 6.0.1 software (Bruker Biospin, RRID: SCR_001964). MRI was performed 1 to 2 weeks after tumor cell implantation and repeated weekly to monitor GBM tumor growth. Mice were anesthetized with 3.5% isoflurane and maintained on 1% to 2% isoflurane during the MRI scan. Mice were positioned head prone for measurements, and breathing was monitored to ensure the well-being of the animals. Tumor visualization and discrimination from surrounding tissue was achieved by combining T1-weighted, T2-weighted, and diffusion-weighted imaging techniques, and apparent diffusion coefficient maps were subsequently calculated (Supplementary Table S2). For determination of tumor volume, tumor tissue was segmented manually in the T2-weighted images. Regions of interest were placed in all slices bearing tumor tissue. Tumor volume in mm³ was calculated from the area represented by the included pixels and multiplied by slice thickness.

### The Cancer Genome Atlas target Genotype-Tissue Expression data mining

The UCSC Xena Functional Genomics Explorer software (RRID: SCR_018938) was used to explore integrin β1, integrin β3, and FAK gene expression profiles in The Cancer Genome Atlas (TCGA) Target Genotype-Tissue Expression (GTEx) database, a compilation of clinical and gene expression data from TCGA compared with normal brain tissue from the GTEx projects (34). Briefly, the TCGA Target GTEx was sorted for expression data localized to the brain which yielded 1,830 cases from healthy normal individuals (GTEx, *n* = 1,141) and from those suffering from lower-grade glioma (TCGA LGG, WHO grades 2 and 3, *n* = 523) or GBM (TCGA GBM, *n* = 166). For those patients with survival statistics, Kaplan–Meier survival curves were generated based on median log_2_(norm_count + 1) gene expression for integrin β1 and integrin β3 within the specified glioma grade. Gene expression above the median were designated as high, whereas expression below the median were considered low; patients were sorted based on β1^High^β3^High^, β1^High^β3^Low^, β1^Low^β3^High^, and β1^Low^β3^Low^ expression. Differentially expressed genes between β1^High^β3^High^ versus β1^Low^β3^Low^ LGG and GBM were subsequently identified by the characteristic direction method and analyzed in the Molecular Signatures Database Hallmark gene sets using Xena gene set enrichment analysis with blitzGSEA (35, 36).

### Statistical analysis

Significance was determined using Student two-tailed *t* tests or one-way ANOVA followed by the post hoc Tukey multiple comparisons test (GraphPad Prism 5, RRID: SCR_002798) with *P* < 0.05 considered significant. Error bars show the mean ± SEM. Kaplan–Meier survival curves with the log-rank test (Mantel–Cox) were used to determine survival statistics.

### Data availability

The data generated in this study are available upon request from the corresponding author.

## Results

### Clotting correlates with GBM progression

**Figure 1:**
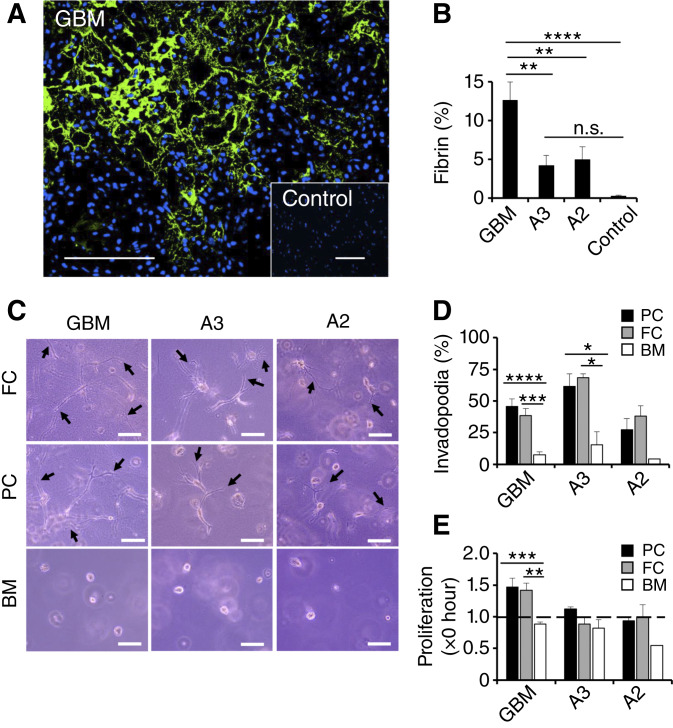
Clotting correlates with GBM progression. **A** and **B,** Fibrin formation was assessed in tumor tissues from patients with astrocytoma grade 2 (A2; *n* = 3), astrocytoma grade 3 (A3; *n* = 3), or GBM (*n* = 3) compared with healthy brain tissue (Control; *n* = 3) by fluorescence microscopy. **A,** Representative images of fibrinogen (green) in GBM and healthy brain tissue are shown. Nuclei are stained with DAPI (blue). Scale bar, 100 μm. **B,** Percentage of fibrin(ogen)-positive areas per optical field. **C–E,** Primary tumor cells freshly isolated from patients with astrocytoma grade 2 (A2, *n* = 2), astrocytoma grade 3 (A3, *n* = 3), or GBM (*n* = 12) were embedded in a 3D matrix of PC, FC, or Matrigel BM. Representative phase-contrast images are shown after 4 days in high magnification. Black arrows show invadopodia. Scale bar, 100 μm (**C**). **D,** Invadopodia-positive tumor cells were counted as percent of total per optical field after 4 days of embedding in PC, FC, or BM. **E,** Proliferation was assessed per optical field as fold increase of cells after 4 days of embedding. The dotted line reflects baseline cell numbers. *, *P* < 0.05; **, *P* < 0.01; ***, *P* < 0.001; ****, *P* < 0.0001. BM, basement membrane; FC, fibrin clot; n.s., nonsignificant; PC, plasma clot.

The prometastatic effects of coagulation factors on circulating tumor cells are well established (30, 31). To study the role of clotting in astrocytoma progression, we probed tumor tissue from patients with GBM as well as astrocytoma grades 2 and 3 compared with normal brain tissue for expression of fibrinogen and its clotting product fibrin. IHC and subsequent fluorescence microscopy revealed a positive correlation between tumor grade and fibrin accumulation in the interstitial spaces of patient tissues, with the highest amounts of fibrin in GBM and lower levels in astrocytoma grades 2 and 3 (Fig. 1A and B; Supplementary Fig. S1). Normal brain tissue was virtually fibrin-free. To assess the functional significance of clotting for glioma progression, we embedded primary tumor cells from patients with GBM as well as astrocytoma grades 2 and 3 in fibrin gels generated from purified fibrinogen (fibrin clot) or blood plasma (plasma clot) compared with Matrigel basement membrane matrix. Scoring the 3D matrices by phase-contrast microscopy after 96 hours for invadopodia-positive cells and overall tumor cell expansion, revealed extensive cell invasion in fibrin and plasma clot for all astrocytoma grades whereas only GBM cells managed to proliferate efficiently under these culture conditions (Fig. 1C–E). Invasion and proliferation in Matrigel basement membrane matrix, on the other hand, was impaired even in GBM cells supplemented with 2.5 U/mL thrombin with or without 3 mmol/L CaCl_2_, suggesting that blood clotting specifically promotes tumor cell functions relating to glioma progression (Supplementary Fig. S2A–S2C).

### Clotting promotes GBM growth *in vivo*

**Figure 2:**
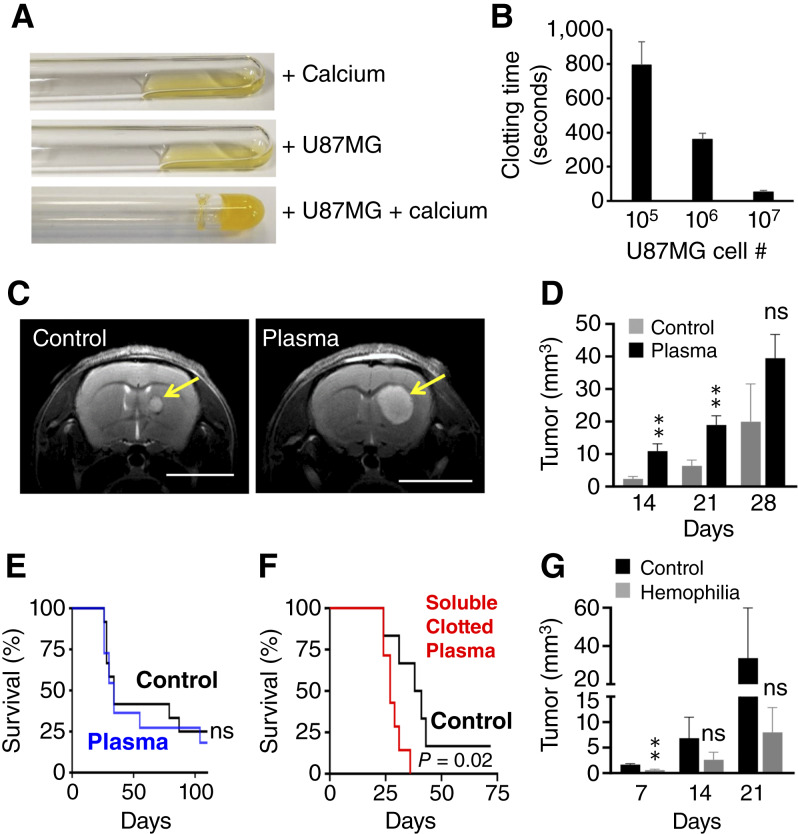
Clotting promotes GBM growth *in vivo*. **A,** Representative images depicting clotting of citrated human blood plasma spiked with U87MG GBM cells in the presence of calcium *in vitro* (bottom), whereas blood plasma mixed with calcium (top) or U87MG cells alone (middle) failed to clot. **B,** Clotting time of plasma after incubation with U87MG cells together with calcium. **C** and **D,** U87MG GBM xenografts were implanted with or without calcified human blood plasma (plasma vs. control; *n* = 6) into the brain of athymic nude mice and monitored by MRI. Representative T2-weighted images after 2 weeks are shown with tumors depicted by yellow arrows. Scale bar, 500 μm (**C**). **D,** Tumor volume was calculated over time. **E,** Kaplan–Meier survival curves of mice bearing U87MG xenographs implanted together with calcified human blood plasma (*n* = 11) or serum-free media (control, *n* = 12) and monitored for 110 days. The median survival time was 34 days for both plasma and control. **F,** Kaplan–Meier survival curve of athymic nude mice implanted with U87MG tumor cells together with the soluble fraction of clotted plasma (*n* = 7) compared with U87MG cells in serum-free media (control, *n* = 6). The median survival time was 27 days for the clotted plasma group and 38 days for the control group. **G,** GL-261 GBM cells were suspended in plain media and implanted into the brain of hemophilia (*n* = 4) and control mice (*n* = 5). Tumor volume was calculated over time by MRI. *, *P* < 0.05; **, *P* < 0.01; ns, not significant.

To further delineate the role of clotting in brain tumors, we suspended cultured GBM cells in citrated human blood plasma and analyzed clotting *in vitro* after addition of 3 mmol/L calcium (Fig. 2A and B). Whereas addition of calcium or tumor cells alone was not sufficient to induce clotting, we observed strong and accelerated clotting after mixing the calcified plasma with human U87MG GBM cells. Although CaCl_2_ was necessary to promote clotting, we did not see an effect on tumor invasion, growth, or survival after adding 3 mmol/L CaCl2 to GBM cells in suspension or embedded in Matrigel (Supplementary Fig. S2). To test if the presence of blood plasma affects U87MG xenograft growth *in vivo*, we suspended 500,000 U87MG cells in a final volume of 5 μL citrated plasma. The mixture of U87MG cells and blood plasma was injected into the brain of athymic nude mice immediately after the addition of calcium and monitored for xenograft formation by MRI on a weekly basis. The MRI scans revealed a significant growth advantage of U87MG cells in blood plasma compared with cell culture media over 3 weeks, which declined toward the end of our measurements after 4 weeks (Fig. 2C and D; Supplementary Fig. S3). Accordingly, coinjection of U87MG cells with blood plasma did not translate into a reduced OS (Fig. 2E). To ensure that the initial tumor growth–promoting effect of the blood plasma was due to clotting activity, we induced clotting in blood plasma *in vitro* and collected the soluble fraction of the clotted plasma. Notably, coinjection of U87MG cells with soluble clotted plasma, which is highly enriched with soluble fibrin and fibrin degradation products, into the brain of athymic nude mice resulted in a significant decrease of survival time compared with U87MG cells suspended in serum-free cell culture media (Fig. 2F). Lastly, we assessed the role of clotting for brain tumor progression in severely clotting-deficient hemophilia A mice by MRI, which revealed delayed tumor growth after intracerebral injection of GL-261 murine GBM cells in hemophilia mice compared with coagulation-competent wild-type mice (Fig. 2G; Supplementary Fig. S3). Taken together, these data indicate that clotting provides an important stimulus for the expansion of GBMs *in vivo*.

### Clotting promotes invasion and growth of GBM cells in a 3D culture model

**Figure 3:**
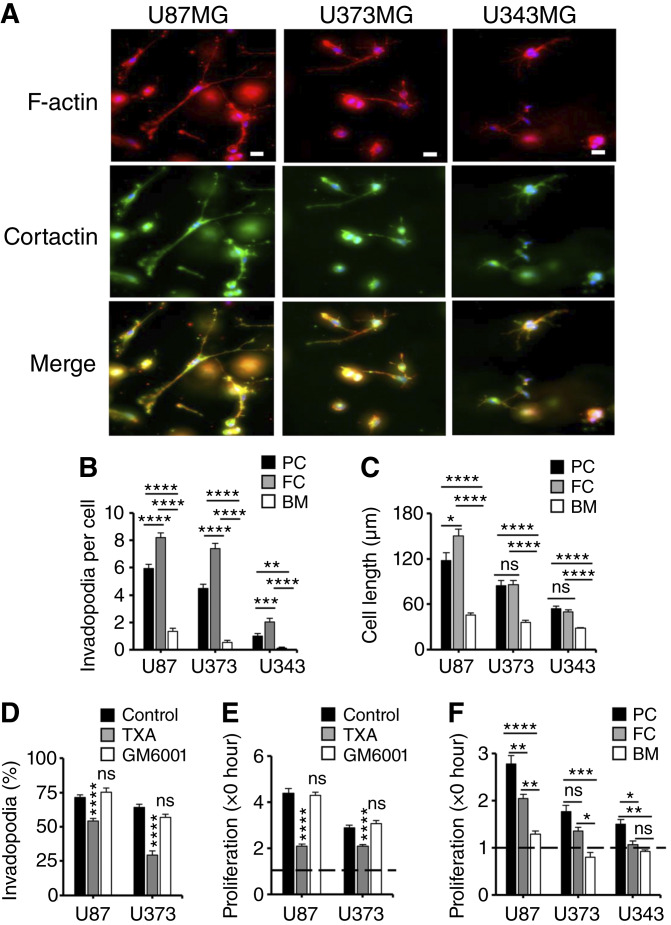
Clotting promotes invasion and growth of GBM cells in a 3D culture model. **A,** U87MG, U373MG, and U343MG cells were fixed 2 days after embedding in fibrin, stained for F-actin (red) and cortactin (green), and analyzed by fluorescence deconvolution microscopy. Representative images are shown. Scale bar, 20 μm. **B,** The cumulative number of invadopodia generated per cell over 4–6 hours in the first 16 hours after embedding in PC, FC, or Matrigel BM is shown. **C,** Cell length was established by measuring the largest diameter per cell after 16 hours. **D** and **E,** Invadopodia formation (**D**) and proliferation (**E**) of fibrin-embedded U87MG and U373MG cells 4 days after treatment with 3 mmol/L TXA or 10 μmol/L of the matrix metalloproteinase inhibitor GM6001 compared with control. **F,** Fold increase in the numbers of U87MG, U373MG, and U343MG cells per optic field after 3 days of embedding in PC, FC, or BM. The dotted line reflects baseline cell numbers.*, *P* < 0.05; **, *P* < 0.01; ***, *P* < 0.001; ****, *P* < 0.0001. BM, basement membrane; FC, fibrin clot; ns, not significant; PC, plasma clot.

An interesting feature of GBM cells cultured in 3D clot *in vitro* is their rapid shape change from round to elongated, which includes the continuous outgrowth and subsequent retraction of extensions that stained strongly positive for the invadopodia components F-actin and cortactin (Fig. 3A). Quantitative analysis of real-time video microscopy images revealed that this process was typical for U87MG and, to a lesser extent, U373MG GBM cells, as the two cell lines generated more and longer invadopodia in fibrin and plasma clot than in Matrigel (Fig. 3B and C; Supplementary Videos S1–S6). U343MG GBM cells, on the other hand, were the least invasive of the three cell lines. Likewise, U87MG cells proved to be most motile, followed by U373MG and U343MG cells (Supplementary Fig. S4A–S4C). Invasion of U87MG and U373MG cells in fibrin clot required the proteolytic activity of plasmin as we found significantly reduced invadopodia formation in response to the antifibrinolytic agent TXA but no effect after treatment with the matrix metalloproteinase inhibitor GM6001 (Fig. 3D). The anti-invasive properties of TXA translated into significant growth inhibition in 3D fibrin, suggesting that a connection between invadopodia formation and cell proliferation exists (Fig. 3E). The correlation between invadopodia formation and proliferation was also apparent after embedding of GBM cells in Matrigel, which exhibited reduced invasion as well as delayed proliferation compared with fibrin or clotted plasma (Fig. 3B, C, and F). Taken together, these experiments suggest that fibrinolysis stimulates invadopodia formation and at the same time provides a growth-promoting microenvironment for GBM cells.

### Invadopodia formation and growth of GBM cells in plasma clot depend on integrins β1 and β3

**Figure 4:**
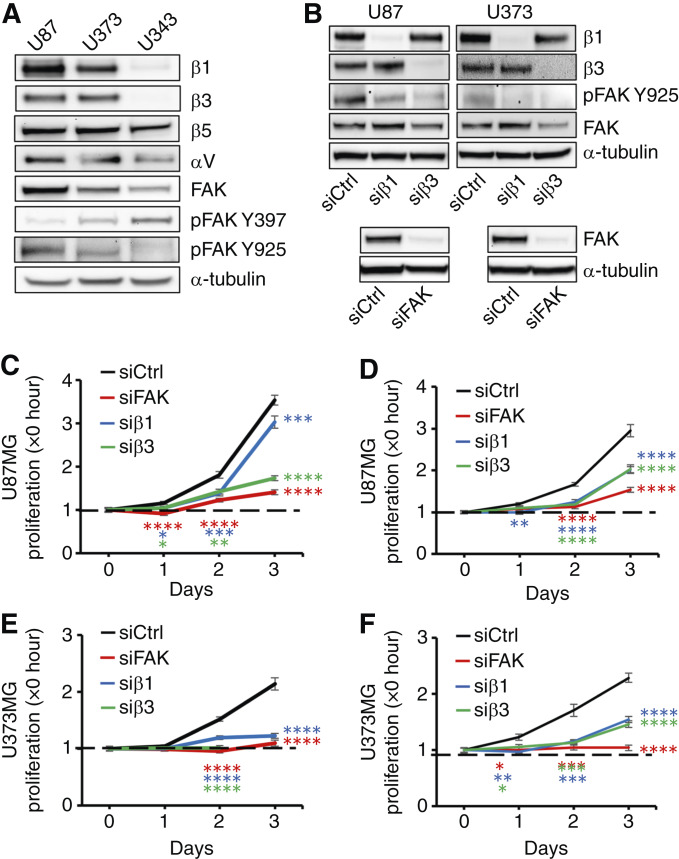
Growth of GBM cells in fibrin clot and plasma clot depends on integrins β1 and β3. **A,** Integrins β1, β3, β5, and αV as well as total and activated FAK at Y397 and Y925 were analyzed in subconfluent extracts from U87MG, U373MG, and U343MG GBM cells by immunoblotting. α-tubulin served as a loading control. **B,** Western blot analysis of integrin β1 and integrin β3 expression and total FAK and FAK activation at Y925 in extracts from U87MG and U373MG cells 3 days after transfection with siRNA against integrins β1 (siβ1) and β3 (siβ3) compared with treatment with control siRNA (siCtrl; **B**, top). FAK expression after transfection with siRNA against FAK (siFAK) compared with siCtrl (**B**, bottom). **C–F,** Fold increase in cell proliferation was analyzed over time in fibrin clot–embedded (**C**) and plasma clot–embedded (**D**) U87MG cells and in fibrin clot–embedded (**E**) and plasma clot–embedded (**F**) U373MG cells following transfection with siβ1, siβ3, and siRNA against FAK compared with siCtrl by phase-contrast microscopy. *, *P* < 0.05; **, *P* < 0.01; ***, *P* < 0.001; ****, *P* < 0.0001 compared with siCtrl.

Invadopodia formation depends on adhesive interactions of integrin cell surface receptors with the ECM (37). To define the adhesive signature of GBMs in clot invasion, we compared integrin expression in U87MG, U373MG, and U343MG cells and recognized that less invasive and proliferative U343MG cells expressed limited amounts of integrin β1 and β3 whereas other integrins such as αv and β5 were fairly evenly expressed among all three GBM cell lines (Fig. 4A). Similarly, we found that primary GBM cells that had reduced expression of both integrins β1 and β3 showed limited invadopodia formation and reduced growth in plasma as well as fibrin clot (Supplementary Fig. S5). Moreover, increased expression of β1 and β3 integrins in U87MG and U373MG cells correlated with higher levels of total FAK (Fig. 4A). Whereas activation of FAK at tyrosine 397 was the strongest in U343MG cells and the weakest in U87MG cells in 2D culture on plastic, we detected the strongest tyrosine 397 activation after embedding U87MG cells in fibrin, suggesting that integrin ligation plays a greater role in 3D clot than on 2D plastic (Fig. 4A; Supplementary Fig. S6). The pattern of FAK activation at tyrosine 925, on the other hand, was the same after embedding in 3D fibrin compared with 2D culture on plastic. Knocking down integrin β1 or β3 in U87MG and U373MG cells with siRNA reduced FAK activation and led to a marked antiproliferative effect in GBM cells embedded in fibrin and plasma clot (Fig. 4B–F). Accordingly, we found a near-complete inhibition of GBM cell proliferation in fibrin as well as plasma clot after knocking down FAK with siRNA. Partial inhibition of invadopodia formation in GBM cells embedded in plasma clot could be achieved with siRNA against integrins β1 and β3 and FAK (Supplementary Fig. S7A and S7B). Invadopodia formation in fibrin, on the other hand, depended on β3 integrin independent of FAK expression (Supplementary Fig. S7C and S7D). Collectively, our data indicate that adhesive interactions of integrins with fibrin and other plasma-derived ligands promote activation of FAK, which in turn mediates the progrowth effects of plasma clot on GBM cells.

### Silencing FAK in GBM cells is antiproliferative and antitumorigenic

**Figure 5:**
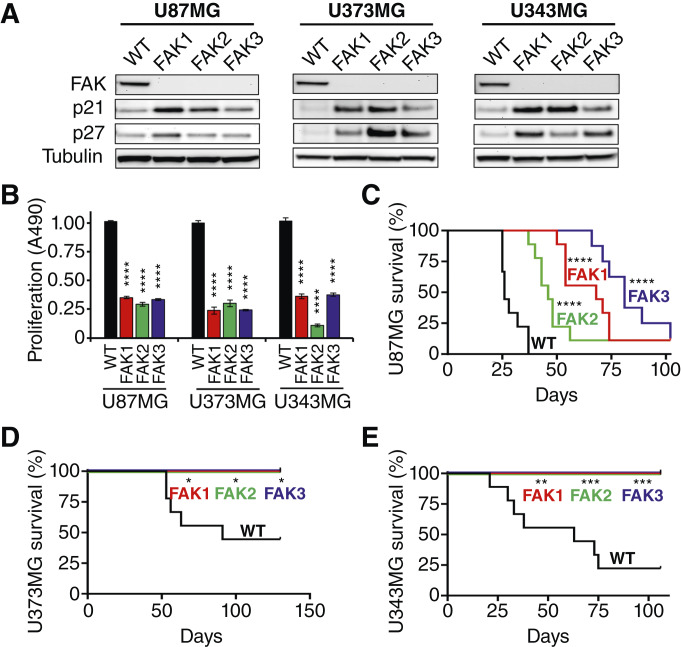
Silencing FAK in GBM cells is antiproliferative and antitumorigenic. **A,** Subconfluent extracts from U87MG (left), U373MG (middle), and U343MG (right) cells treated with FAK CRISPR-cas9 (three clones each) were probed for the expression of FAK and the cell-cycle regulators p21 and p27 by immunoblotting. Wild-type (WT) parental cells served as controls. α-tubulin served as a loading control. **B,** Proliferation of FAK knockout GBM cells (FAK 1–3) after 4 days compared with WT cells. WT proliferation was set to 1.0. **C,** Kaplan–Meier survival curves of athymic nude mice intracranially injected with U87MG FAK KO cells (FAK 1–3) in serum-free media compared with U87MG WT. Survival was monitored for 102 days. Median survival time was 26 (WT), 68 (FAK-1), 46 (FAK-2), and 81 (FAK-3) days after tumor cell injection (*n* = 8–9). **D** and **E,** Kaplan–Meier survival curves after intracranial injection of U373MG (**D**) and U343MG (**E**) WT and FAK knockout cells (FAK 1–3) into athymic nude mice. Median survival time was 91 (U373 WT) and 63 (U343 WT) days after tumor cell injection, with no death occurring in mice implanted with the FAK KO clones during 130 and 106 days, respectively (*n* = 8–9). *, *P* < 0.05; **, *P* < 0.01; ***, *P* < 0.001; ****, *P* < 0.0001 vs. WT.

As FAK seems to have an important role in transmitting progrowth signals of GBM cells downstream of integrins β1 and β3, we permanently silenced FAK in U87MG, U373MG, and U343MG cells using CRISPR-Cas9. Following the gene editing procedure, we randomly selected three clones from each cell line that lacked detectable FAK protein expression for further testing (Fig. 5A). Knocking out FAK coincided with upregulation of the cell-cycle inhibitors p21 and p27 in each of the three U87MG, U373MG, and U343MG clones compared with the respective parent GBM, resulting in a sustained growth reduction under standard cell culture conditions (Fig. 5A and B). The antiproliferative effect of FAK silencing in GBM cell lines *in vitro* translated into a significantly increased survival of up to 81 days after implantation of U87MG-FAK-KO cells into the brains of athymic nude mice compared with U87MG parental cells *in vivo* (Fig. 5C). Moreover, there was no detectable tumor formation after implantation of U373MG-FAK-KO and U343MG-FAK-KO cells into athymic nude mice, which did not exhibit any signs of GBM-related illness during the observation period (Fig. 5D and E). We, therefore, conclude that targeting FAK in GBM cells reduces tumor cell proliferation and may impair tumorigenicity in malignant brain tumors.

### Integrin expression in gliomas affects patient outcome

**Figure 6:**
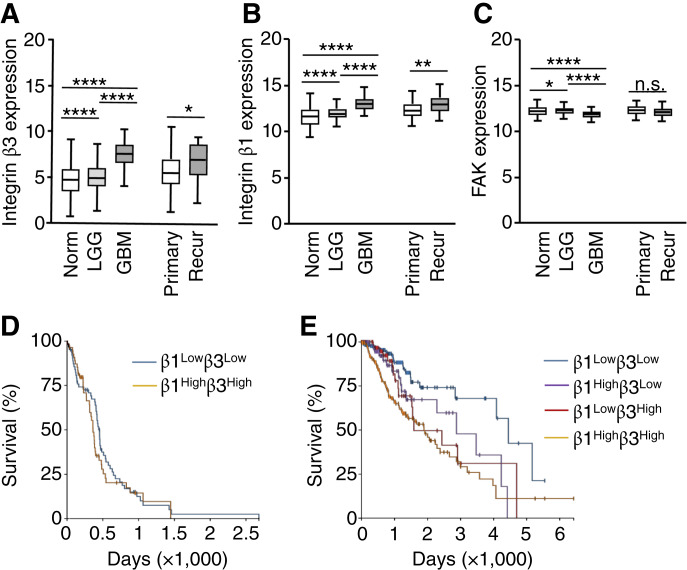
Expression of integrin β1 and integrin β3 affects patient outcome. **A–C,** Left, Integrin β3 (**A**), integrin β1 (**B**) and FAK (**C**) mRNA levels in healthy normal brain tissue (*n* = 1,141) compared with tumor tissue isolated from patients with LGG (WHO grades 2 and 3, *n* = 523) or GBM (*n* = 166). Values are expressed as log_2_(norm_count+1) with the median (line), range (box), and SD (bars) indicated for each group. **A–C,** Right, Integrin β3 (**A**), integrin β1 (**B**), and FAK (**C**) mRNA expression in patients with primary tumors (primary, *n* = 662) compared with recurrent disease (*n* = 27). **D,** Kaplan–Meier survival curve for 165 patients with GBM sorted based on median integrin β1 [13.2 log_2_(norm_count + 1)] and integrin β3 [7.62 log_2_(norm_count + 1)] expression for tumors with high expression of both integrins above the median (β1^High^β3^High^, *n* = 55) or low expression of both integrins below the median (β1^Low^β3^Low^, *n* = 63). Log-rank test statistics = 0.6787, *P* = 0.41. **E,** Kaplan–Meier survival curves for 521 patients with LGG glioma were sorted based on median integrin β1 [12.1 log_2_(norm_count + 1)] and integrin β3 [5.01 log_2_(norm_count + 1)] expression to generate four subgroups: β1^Low^β3^Low^ (*n* = 186), β1^High^β3^Low^ (*n* = 73), β1^Low^β3^High^ (*n* = 66), and β1^High^β3^High^ (*n* = 196). β1^High^β3^High^ vs. β1^Low^β3^Low^, log-rank test statistics = 25.32, *P* < 0.0001; β1^High^β3^High^ vs. β1^Low^β3^High^, log-rank test statistics = 3.638, *P* = 0.056; β1^High^β3^High^ vs. β1^High^β3^Low^, log-rank test statistics = 2.727, *P* = 0.099; β1^High^β3^Low^ vs. β1^Low^β3^Low^, log-rank test statistics = 5.207, *P* = 0.02; β1^Low^β3^High^ vs. β1^Low^β3^Low^, log-rank test statistics = 4.875, *P* = 0.03; and β1^High^β3^Low^ vs. β1^Low^β3^High^, log-rank test statistics = 0.03, *P* = 0.87. Norm, normal; Recur, recurrent.

To assess the clinical relevance of glioma adhesion to the ECM, we analyzed expression of integrin β1, integrin β3, and FAK in a cohort of patients with glioma using data from TCGA and the GTEx projects (TCGA LGG, WHO grades 2 and 3, *n* = 523; TCGA GBM, *n* = 166; GTEx normal brain tissue, *n* = 1,141) in conjunction with the Xena bioinformatics tool from University of California Santa Cruz (34). Our analysis of the publicly accessible data bases revealed a marked upregulation of integrin β3 mRNA in GBM tissues compared with LGG and healthy brain tissue (2.6 log_2_-fold and 2.9 log_2_-fold, respectively) as well as in recurrent versus primary gliomas (1.5 log_2_-fold; Fig. 6A). Integrin β1 and FAK, on the other hand, were broadly expressed in normal brain and LGG, with integrin β1 being upregulated in GBM and recurrent glioma whereas FAK was slightly downregulated (Fig. 6B and C). The pattern of pronounced upregulation of integrin β3 in GBM and consistent overall expression of integrin β1 and FAK in all grades of gliomas was reiterated in the Western blot analysis of primary tumor cells resected from patients with glioma at the Saarland University Hospital, indicating a fair amount of overlap between gene expression in glioma tissue and protein expression in isolated glioma cells (Supplementary Fig. S5A). Consistent with these findings, we found downregulation of integrins β1 and β3 in LGG and to a lesser extent in GBM with IDH1 mutation and upregulation of integrins β1 and β3 in EGFR-mutated LGG (Supplementary Fig. S8). To further analyze whether integrin expression correlates with activation of specific glioma functions, we performed gene set enrichment analysis in patients with glioma whom we classified as integrin β1/β3–high or –low when the respective mRNA expression in GBM and LGG was above or below the median, as depicted in Fig. 6A and B. Interestingly, upregulation of integrins β1 and β3 in both GBM and LGG strongly correlates with the activation of pathways involved in EMT, inflammatory response, and, notably, coagulation, suggesting that the tumor microenvironment at large and the clotting system in particular generate critical cues for glioma progression (Table 2). To determine if the activation of these pathways correlates with patient outcome, we assessed OS in patients with glioma whom we classified according to their mRNA expression as integrin β1/β3–high or –low. Whereas the expression levels of these integrins had no bearing on the survival of patients with GBM, the analysis revealed a significantly reduced OS of patients with glioma WHO grades 2 and 3 that overexpressed both integrins β1 and β3 compared with patients with LGG who overexpressed only one of the two integrins or none (Fig. 6D and E). Based on these data, we conclude that adhesive interactions of glioma cells with their respective ligands in the tumor ECM have important implications for glioma progression.

**Table 2:** Top 10 Molecular Signatures Database hallmark gene sets significantly overexpressed in the GBM (A) or LGG (B) groups. β1^High^β3^High^ patient subgroups were compared with the β1^Low^β3^Low^ subgroups

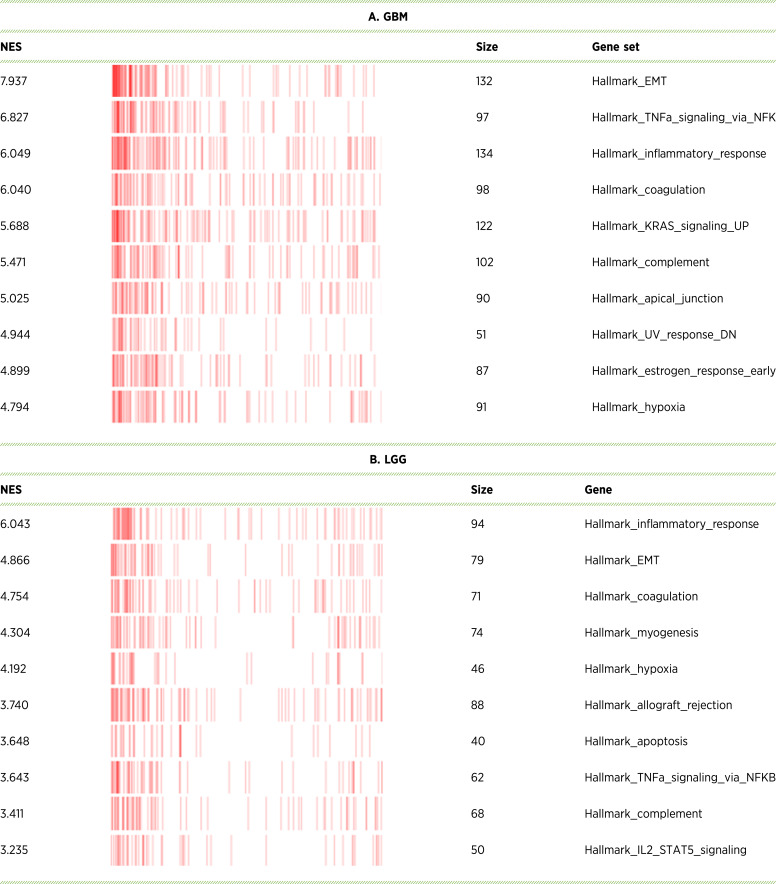

Running sum blot with the distribution of hits relative to the gene ranking within the signature. Size refers to the gene set size.

Abbreviations: NES, normalized enrichment score; UP, upregulated; DN, downregulated.

## Discussion

We previously demonstrated that clot formation promotes lung metastasis (30, 31) and now set out to define the role of clotting for the progression of malignant brain tumors. To this end, we were able to show that the prothrombotic state of gliomas is associated with the generation of a fibrinogen-rich edema and subsequent formation of fibrin in tumor interstitial spaces and that this process is most extensive in tissues from patients with GBM. Clotted plasma, which consists of fibrin and plasma adhesion proteins associated with fibrin, supports sprouting of patient-derived glioma cells *in vitro* and GBM expansion in murine orthotopic tumor models *in vivo*. The capacity of GBM cells to colonize clotted plasma depends on adhesive interactions with integrins β1 and β3 that result in activation of FAK, thereby providing critical cues for glioma proliferation *in vitro* and glioma progression *in vivo*.

Malignant tumor cells typically promote clotting due to increased expression of procoagulant and antifibrinolytic factors (23, 24). The resulting state of hypercoagulability is particularly prominent in patients with GBM as their course of disease is frequently complicated by the occurrence of deep vein thrombosis and venous thromboembolism (ref. 38). Venous thromboembolism in patients with GBM often coincides with thrombotic occlusion of the tumor vasculature, which explains the development of tumor cell necrosis commonly observed in GBM (28, 39). The driving force behind the prothrombotic state is the overexpression of tissue factor in high-grade gliomas, which is further enhanced by the hypoxic conditions in areas of tumor ischemia (24). Hypoxia is also a strong inducer of VEGF, which due to its unfettered release in GBMs leads to the formation of a hyperplastic and distorted vasculature that is unable to maintain a directed blood flow (40, 41). In addition, tumor blood vessels in GBMs are notoriously leaky (27). The combination of deregulated angiogenesis, hypercoagulability, and subsequent ischemia provides the functional basis for the large deposits of fibrinogen and fibrin that we identified in tumor tissue sections from patients with GBM. Tumor tissues from patients with astrocytoma grades 2 and 3, on the other hand, expressed significantly less fibrin whereas normal brain was essentially free of fibrin. Therefore, our data indicate that the presence of fibrin in the tumor interstitial spaces is a specific modification of the ECM of malignant brain tumors. This notion that the prothrombotic state of gliomas is associated with poor outcome was reinforced by the finding that mutant IDH1 confers potent antithrombotic properties that prevent patients with IDH1-mutated gliomas from experiencing venous thromboembolism, intratumoral thrombosis, and tumor necrosis (29).

Our data indicate that glioma cells receive critical signals from plasmatic clotting factors in the ECM that stimulate invasion and proliferation *in vitro* and orthotopic tumor growth *in vivo*. As such, we detected a significant acceleration of xenograft formation when we injected U87MG GBM cells together with blood plasma into the brain of immunodeficient mice. This result was confirmed by our finding of delayed GBM growth in transgenic hemophilia A mice, which harbor a severe clotting deficit based on a defect in the gene of coagulation factor VIII (33). The effect of clotting on GBM expansion was greatest in the early phase after tumor cell injection, suggesting similarities to circulating tumor cells in which fibrin provides a transitional ECM until tumor cells are able to organize the microenvironment on their own (30). The subsequent turnover of the clot matrix by fibrinolysis is an important protumorigenic feature in this context, as soluble clot fragments have been shown to induce integrin activation (31). Accordingly, GBM growth in fibrin *in vitro* was markedly inhibited after treatment with the fibrinolysis inhibitor TXA, and brain tumor growth *in vivo* was strongly promoted after coinjecting GBM cells with the soluble fraction of clotted plasma that was enriched with monomeric fibrin and fibrin degradation products. Therefore, our data indicate that the formation and subsequent degradation of blood clot is an important factor for the expansion of gliomas *in vitro* and *in vivo*.

GBM cell lines are known to exhibit considerable differences in genetics and growth compared with patient GBMs. In the present study, we were able to show that the permissive function of blood clotting seen in established cell lines also extended to primary tumor cells isolated from patients with GBM. The primary GBM cells readily infiltrated 3D matrices of plasma clot *in vitro* followed by sustained cell proliferation. The basement membrane mixture Matrigel, on the other hand, was less effective in mediating glioma growth and infiltration, suggesting that fibrin and its degradation products make specific contributions to glioma progression. Effective infiltration and proliferation was best represented in U87MG GBM cells, which overexpress adhesion receptors of the β1 and β3 integrin family. Binding of β1 and β3 integrins to specific sites in fibrin and presumably other clot-associated glycoproteins such as fibronectin induced the formation of invadopodia, which we classified based on the incorporation of F-actin and cortactin (42). In addition, inhibitory experiments with TXA demonstrated that invadopodia formation in fibrin required the fibrinolytic activity of plasmin (43). The anti-invasive effect of TXA translated into growth inhibition in fibrin-embedded GBM cells, suggesting that invasion and proliferation of GBM cells are supported by overlapping adhesive functions. Accordingly, we detected significant inhibition of invasion and growth of GBM cells in fibrin as well as plasma clot after knocking down integrins β1 and β3. A detailed analysis of these assays revealed a strong dependence on integrin β3 for glioma invasion and growth in fibrin. However, invasion and growth in clotted plasma, which contains fibronectin and vitronectin in addition to fibrin, seems to be promoted by β1 as well as β3 integrin. This redundancy between integrins β1 and β3 could be one of the reasons for the treatment failure of cilengitide in patients with GBM and prompted us to look into FAK as a common downstream target of integrins β1 and β3 (44, 45).

Our data suggest that integrins β1 and β3 stimulate GBM growth in plasma clot through activation of FAK, which becomes phosphorylated at tyrosine 397 following adhesion to the fibrin matrix. Integrin-mediated activation of FAK at tyrosine 397 leads to recruitment of Src kinase and subsequent activation of critical intracellular signaling pathways such as activation of PI-3K/Akt and p44/42 MAPK (46, 47). This signaling cascade seems to be relevant for infiltrating brain tumors as FAK has been shown to be upregulated in glioma compared with more benign brain tumors, and overexpression of FAK in GBM cells resulted in increased growth of orthotopic xenografts in immunodeficient mice (48, 49). Our tests demonstrate that knocking down integrins β1 and β3 with siRNA leads to inhibition of FAK activation and that knocking down FAK with siRNA has a strong antiproliferative effect on GBM cells embedded in fibrin as well as clotted plasma. Moreover, silencing of FAK with CRISPR-Cas9 in GBM cells resulted in a sustained growth inhibition *in vitro* and marked antitumor effects in mice *in vivo*. Subsequent Western blot analysis revealed constitutive upregulation of the cyclin-dependent kinase inhibitors p21^CIP1^ and p27^Kip1^, suggesting that GBM cell adhesion to ligands prominently expressed in blood clot is crucial for cell-cycle progression. The central role of the adhesive machinery in glioma progression was reiterated by mining data from TCGA and the GTEx projects, which showed stage-dependent upregulation of integrins β1 and β3 in the most aggressive glioma subtypes and downregulation in less aggressive forms such as IDH1-mutated gliomas, suggesting that FAK phosphorylation may be upregulated in aggressive and suppressed in less aggressive glioma (34). Notably, high β1 and β3 integrin expression in glioma correlates with upregulation of pathways involved in EMT, inflammation, and coagulation, thereby highlighting the functional connection between glioma invasion, the microenvironment, and the ECM architecture. This connection became apparent in gliomas of all grades and was particularly significant in LGGs in which upregulation of integrins β1 and β3 is associated with significantly reduced patient survival. Therefore, we conclude that adhesive interactions of glioma cells with the tumor ECM in general and clot deposits in particular could make important contributions to glioma progression. Further research into clotting in gliomas and the subsequent integrin-mediated activation of FAK could lay the foundation for novel diagnostic tools and therapeutic targets.

## Supplementary Material

Supplementary Table 2 Detailed MRI settings

Supplementary Fig. 1 Expression of fibrin(ogen) and its clotting product fibrin in glioma tumor tissues

Supplementary Fig. 2 Effect of thrombin and CaCl2 on glioblastoma growth in suspension or embedded in 3D matrigel.

Supplementary Fig. 3 Representative MR images after 2 weeks of glioma grown in mice

Supplementary Fig. 4 U87MG, U373MG and U343MG cells were embedded in a 3-dimensional matrix of fibrin and monitored over 3 days using real-time video microscopy

Supplementary Fig. 5 Expression of integrin β1 and integrin β3 affects invadopodia formation and growth of primary glioblastoma cells in fibrin clot and plasma clot

Supplementary Fig. 6 FAK activation in glioblastoma cells embedded in fibrin clot

Supplementary Fig. 7 Invadopodia formation of GBM cells in fibrin clot and plasma clot depends on integrins β1 and β3

Supplementary Fig. 8 Expression of Integrin β1 and integrin β3 in gliomas based on IDH1 and EGFR mutation status

Supplementary Video Legend Legend for supplementary videos 1-6

Supplementary Movie_1 U87MG cells on day 1 after embedding in fibrin

Supplementary Movie_2 U87MG cells on day 2 after embedding in fibrin

Supplementary Movie_3 U87MG cells on day 3 after embedding in fibrin

Supplementary Movie_4 U373MG cells on day 1 after embedding in fibrin

Supplementary Movie_5 U343MG cells on day 1 after embedding in fibrin

Supplementary Movie_6 U87MG cells on day 1 after embedding in matrigel

Supplementary Table 1 Clinicopathologic data of tissue donors
