# Supplementary material for: Clotting Promotes Glioma Growth and Infiltration Through Activation of Focal Adhesion Kinase
Source: Cancer Res Commun. 2024 Dec 13;4(12):3124–36. doi: 10.1158/2767-9764.CRC-24-0164 (PMC11638908; doi:10.1158/2767-9764.CRC-24-0164)
Supplement: Supplementary Table 2 — Detailed MRI settings [file crc-24-0164_supplementary_table_2_suppst2.docx]

**Supplementary Table 2** Detailed MRI settings

| Sequence | Resolution [mm] | TR  [ms] | TE  [ms] | NA | FA | ST [mm] | Purpose |
| --- | --- | --- | --- | --- | --- | --- | --- |
| GRE multi silce | 0.13 x 0.13 | 15 | 3.0 | 1 | 10° | 1.0 | Localizer |
| MGE | 0.5 x 0.5 | 20 | 1.5/5.4 | 1 | 30° | 0.5 | Field map for shimming |
| MSME | 0.078 x 0.078 | 800 | 8.0 | 4 | 90°/180° | 0.75 | T1-weighted morphologic imaging |
| Turbo RARE | 0.078 x 0.078 | 2500 | 30.0 | 4 | 90°/180° | 0.75 | T2-weighted morphologic discrimination tumor- adjacent tissue |
| DWI-EPI | 0.078 x 0.078 | 2750 | 19.0 | 3 | 90° | 0.75 | Diffusion-weighted imaging, calculation of ADC maps, differentiation tumor- adjacent tissueGRE |

GRE Gradient Recalled Echo

MGE Multi Gradient Echo 3D

MSME Multi Slice Mutli Echo

Turbo RARE Rapid Acquisition Relaxation Enhancement with flip back (RARE factor 8)

DWI-EPI Diffusion trace Echo planar Imaging; DWI was performed with 4 separate experiments in x, y and z directions each with the diffusion gradients set to 100, 250, 500, 1000s/mm². For each voxel, ADC were calculated from the individual signal intensity from the different diffusion experiments by linear regression with the slope (based on Levenberg-Marquardt)

TR Repetition time

TE Echo time

NA Number of averages

FA Flip angle

ST Slice thickness
