# Supplementary material for: Clotting Promotes Glioma Growth and Infiltration Through Activation of Focal Adhesion Kinase
Source: Cancer Res Commun. 2024 Dec 13;4(12):3124–36. doi: 10.1158/2767-9764.CRC-24-0164 (PMC11638908; doi:10.1158/2767-9764.CRC-24-0164)
Supplement: Supplementary Fig. 1 — Expression of fibrin(ogen) and its clotting product fibrin in glioma tumor tissues [file crc-24-0164_supplementary_fig.1_suppsf1.pdf]

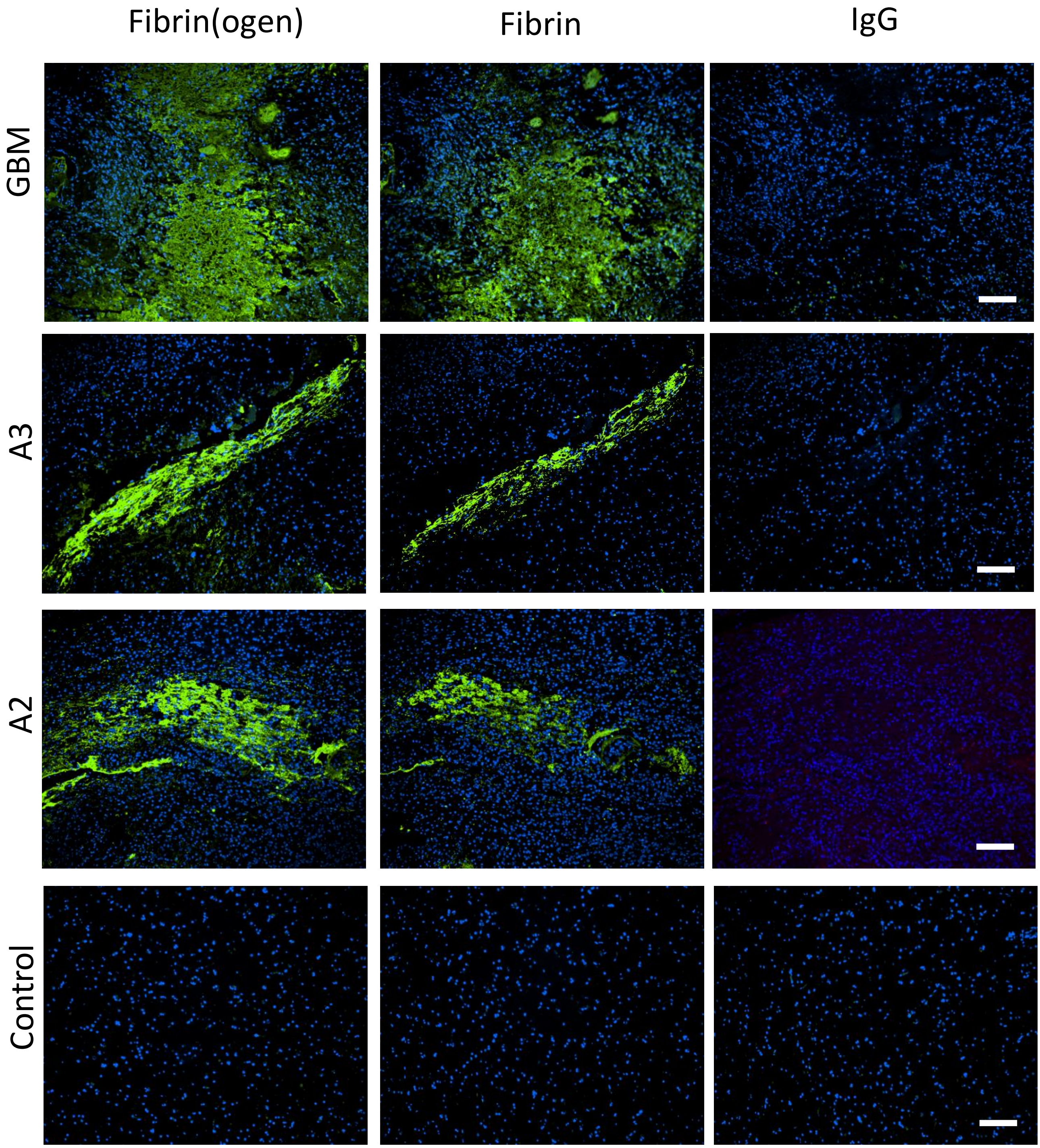

Knowles et al. Supplementary Fig. 1

Supplementary Fig. 1 *Expression of fibrin(ogen) and its clotting product fibrin in glioma tumor tissues.* Expression in tumor tissues from patients with astrocytoma grade 2 (A2), astrocytoma grade 3 (A3) or glioblastoma (GBM) compared to healthy brain tissue (Control) was determined by fluorescence microscopy using antibodies against fibrin(ogen)(Nordic-Mubio, catalog # GAHu/Fbg/7S) and fibrin (Sigma-Aldrich, catalog # ZMS1211). Antibody specificity was confirmed using isotype controls (Thermo Fisher Scientific, catalog # 31245; Stem Cell Technologies, catalog #60070). Representative images of fibrin(ogen) expression (green, left image), fibrin expression (green, middle image) and IgG (right image) in GBM, A3, A2 and healthy brain tissue are shown. Nuclei are stained with DAPI (blue). Scale bar, 100 µm.
