# Supplementary material for: Clotting Promotes Glioma Growth and Infiltration Through Activation of Focal Adhesion Kinase
Source: Cancer Res Commun. 2024 Dec 13;4(12):3124–36. doi: 10.1158/2767-9764.CRC-24-0164 (PMC11638908; doi:10.1158/2767-9764.CRC-24-0164)
Supplement: Supplementary Fig. 2 — Effect of thrombin and CaCl2 on glioblastoma growth in suspension or embedded in 3D matrigel. [file crc-24-0164_supplementary_fig.2_suppsf2.pdf]

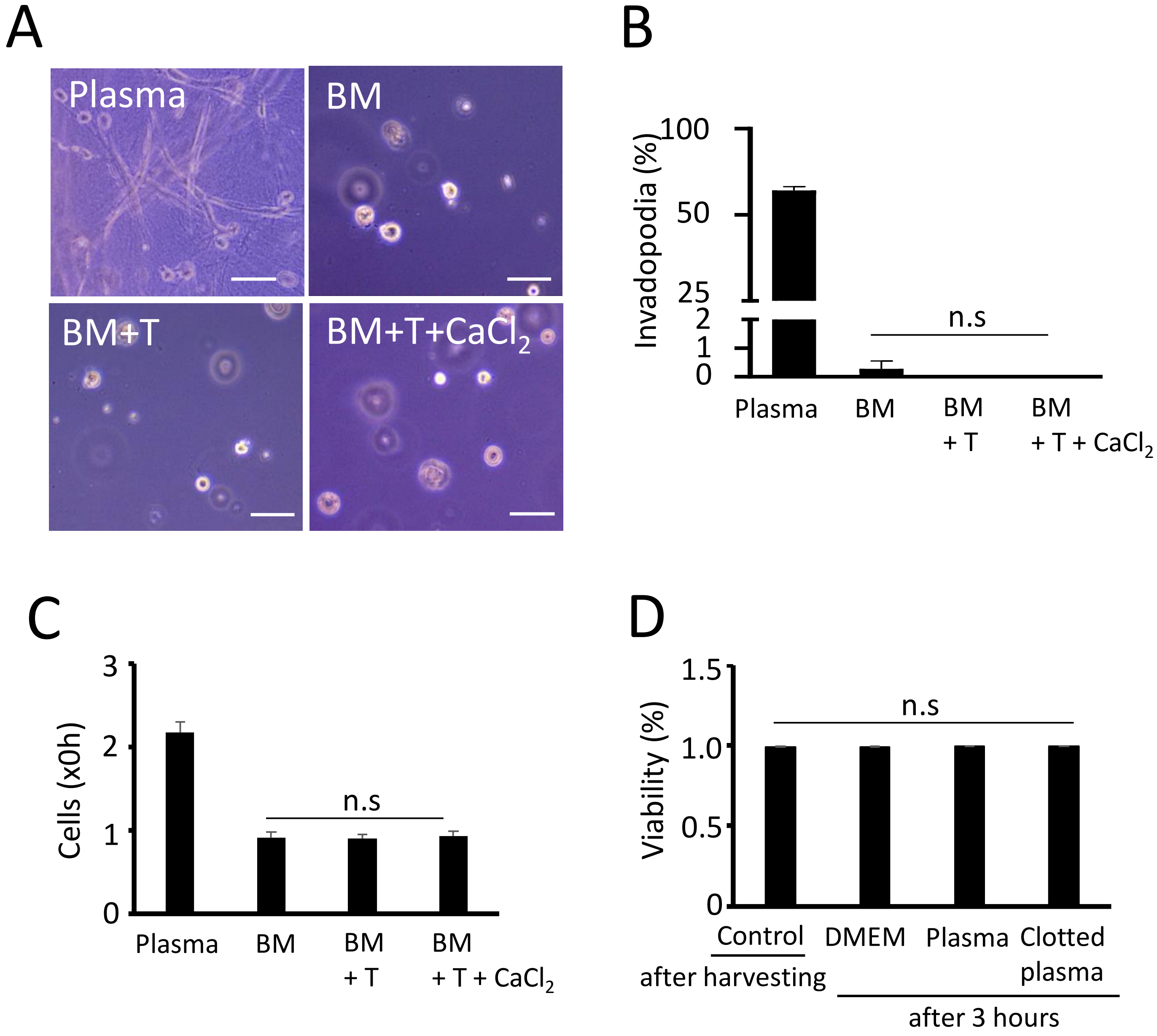

Knowles et al. Supplementary Fig. 2

Supplementary Fig. 2 *Effect of thrombin and $CaCl_2$ on glioblastoma growth in suspension or embedded in 3D matrigel.* (A-C), primary glioblastoma tumors cells isolated from patient 10 were embedded in plasma clot (3.8 % citrated human blood plasma clotted by the addition of 2.5 U/ml thrombin + 3 mM $CaCl_2$) or clots made from matrigel™ basement membrane matrix (BM) solidified at 37°C in the presence or absence of 2.5 U/ml thrombin ± 3 mM $CaCl_2$. All clots were incubated in DMEM media supplemented with 10% FBS for 4 days. Representative phase contrast images are shown (A). Scale bar, 100 μm. Gel plugs were scored for invadopodia (B) and proliferation (C). n.s., non-significant. (D), the viability of U87MG glioblastoma cells was monitored by trypan blue exclusion in cells immediately after harvesting and 3 hours following incubation in DMEM media, human blood plasma (Plasma) or the soluble fraction of clotted plasma containing 3 mM $CaCl_2$ that was generated by contact activation of blood plasma in vitro (Clotted plasma).
