# Supplementary material for: Clotting Promotes Glioma Growth and Infiltration Through Activation of Focal Adhesion Kinase
Source: Cancer Res Commun. 2024 Dec 13;4(12):3124–36. doi: 10.1158/2767-9764.CRC-24-0164 (PMC11638908; doi:10.1158/2767-9764.CRC-24-0164)
Supplement: Supplementary Fig. 3 — Representative MR images after 2 weeks of glioma grown in mice [file crc-24-0164_supplementary_fig.3_suppsf3.pdf]

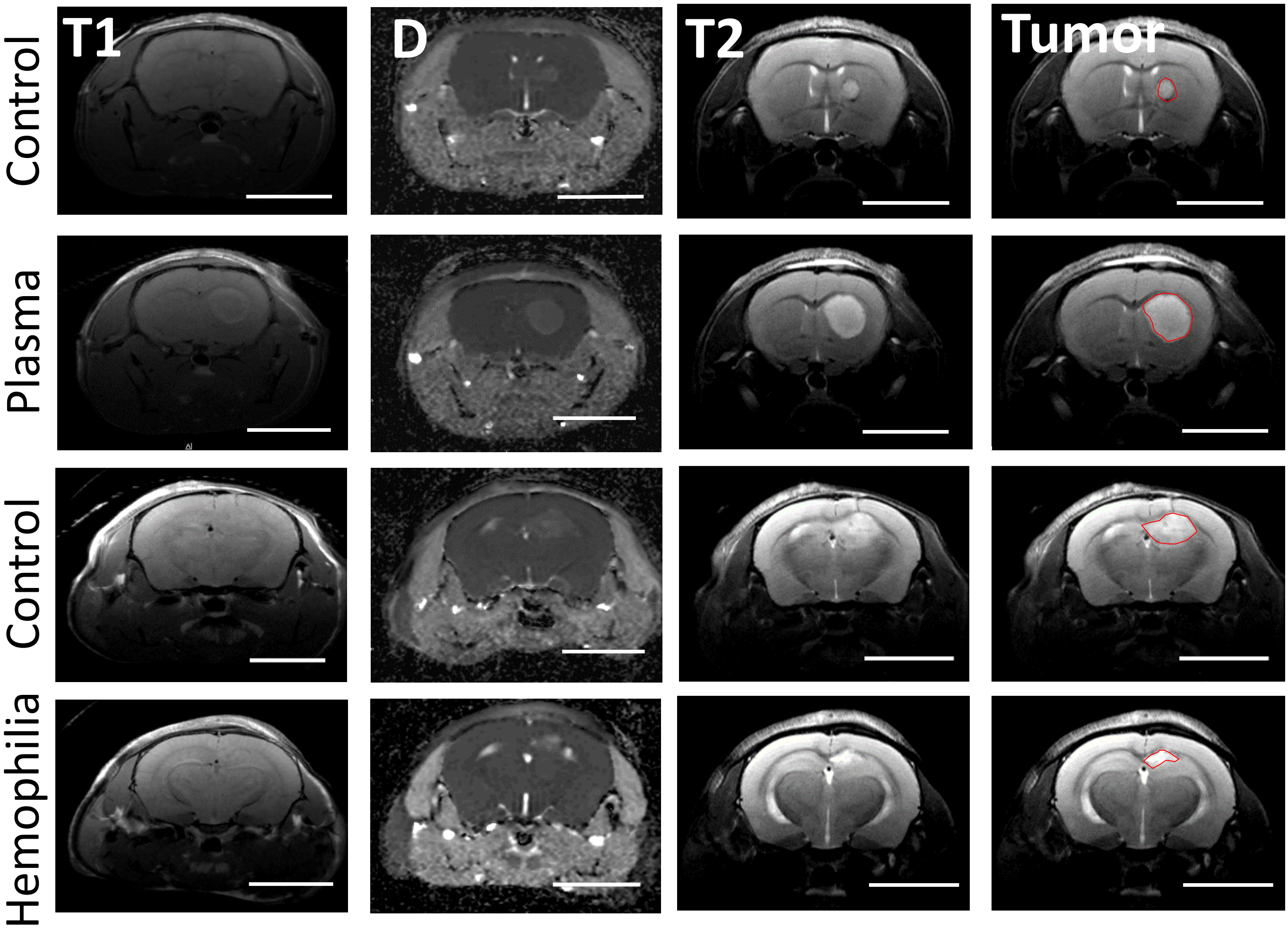

Knowles et al. Supplementary Fig. 3

Supplementary Fig. 3 *Representative MR images after 2 weeks of glioma grown in mice.* T1-weighted (T1, left), diffusion-weighted (D, middle left), T2-weighted (T2, middle right) and T2-weighted images with the tumor area circled in red (Tumor, right) are shown. Either U87MG cells were injected with Control media (1st row) or with calcified human blood plasma (2nd row) in athymic nude mice or GL-261 cells were injected in control (3rd row) or hemophilia A mice (4th row). The scale bar is 500µm.
