# Supplementary material for: Clotting Promotes Glioma Growth and Infiltration Through Activation of Focal Adhesion Kinase
Source: Cancer Res Commun. 2024 Dec 13;4(12):3124–36. doi: 10.1158/2767-9764.CRC-24-0164 (PMC11638908; doi:10.1158/2767-9764.CRC-24-0164)
Supplement: Supplementary Fig. 4 — U87MG, U373MG and U343MG cells were embedded in a 3-dimensional matrix of fibrin and monitored over 3 days using real-time video microscopy [file crc-24-0164_supplementary_fig.4_suppsf4.pdf]

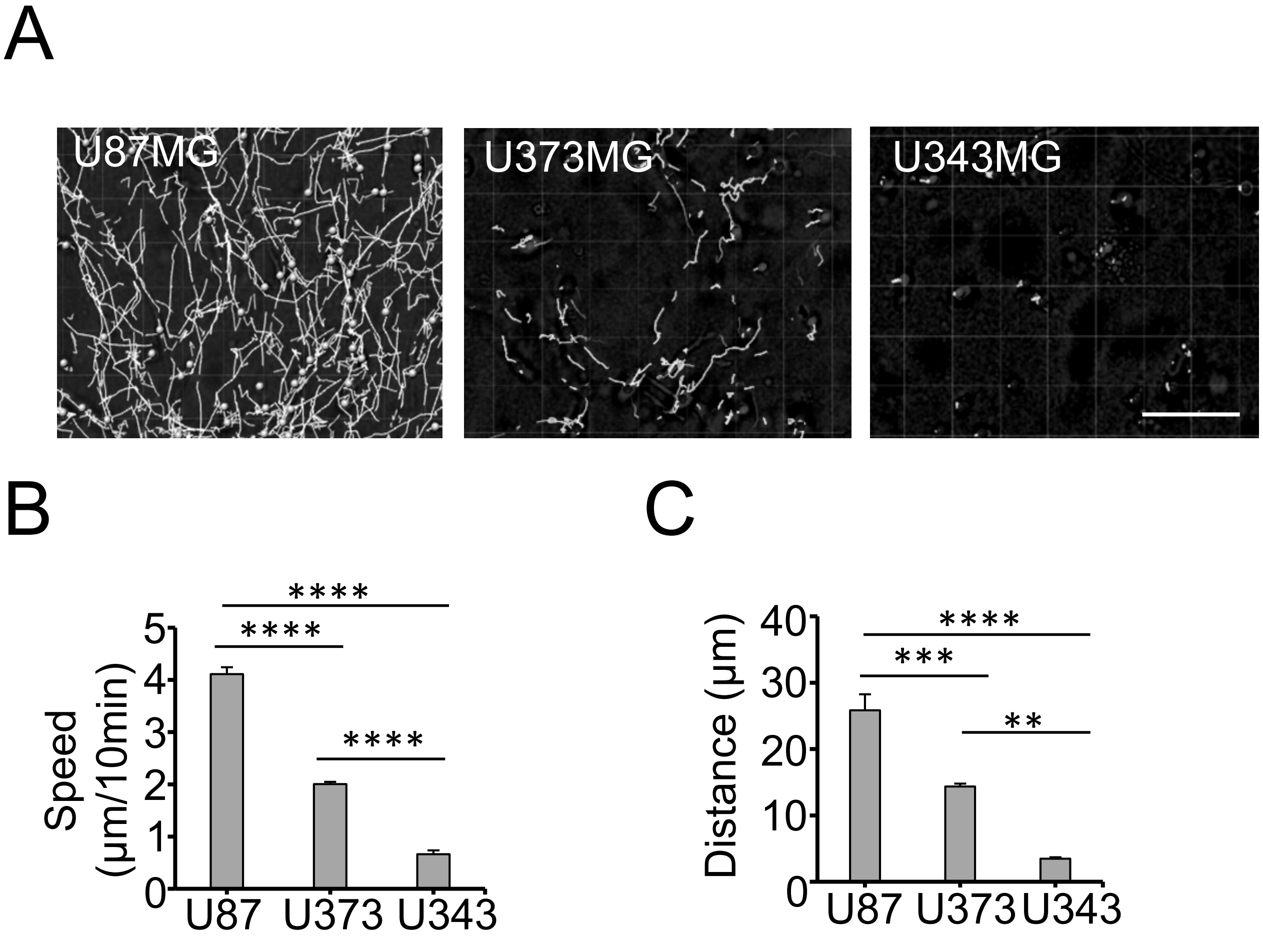

Knowles et al. Supplementary Fig. 4

Supplementary Fig. 4 *U87MG, U373MG and U343MG cells were embedded in a 3-dimensional matrix of fibrin and monitored over 3 days using real-time video microscopy.* (A), representative images depicting cell movement during the observation period are shown. Scale bar, 100 µm. (B-C), speed (B) and distances (C) traveled by U87MG, U373MG and U343MG tumor cells embedded in fibrin clot were calculated using Imaris software 9.8 (Oxford Instruments). Single cells were tracked by the software and processed using an autoregressive motion algorithm. Values for mean track speed and track length were analyzed between day 2 and day 3. **, $P < 0.01$; ***, $P<0.001$; ****, $P < 0.0001$.
