# Supplementary material for: Clotting Promotes Glioma Growth and Infiltration Through Activation of Focal Adhesion Kinase
Source: Cancer Res Commun. 2024 Dec 13;4(12):3124–36. doi: 10.1158/2767-9764.CRC-24-0164 (PMC11638908; doi:10.1158/2767-9764.CRC-24-0164)
Supplement: Supplementary Fig. 5 — Expression of integrin β1 and integrin β3 affects invadopodia formation and growth of primary glioblastoma cells in fibrin clot and plasma clot [file crc-24-0164_supplementary_fig.5_suppsf5.pdf]

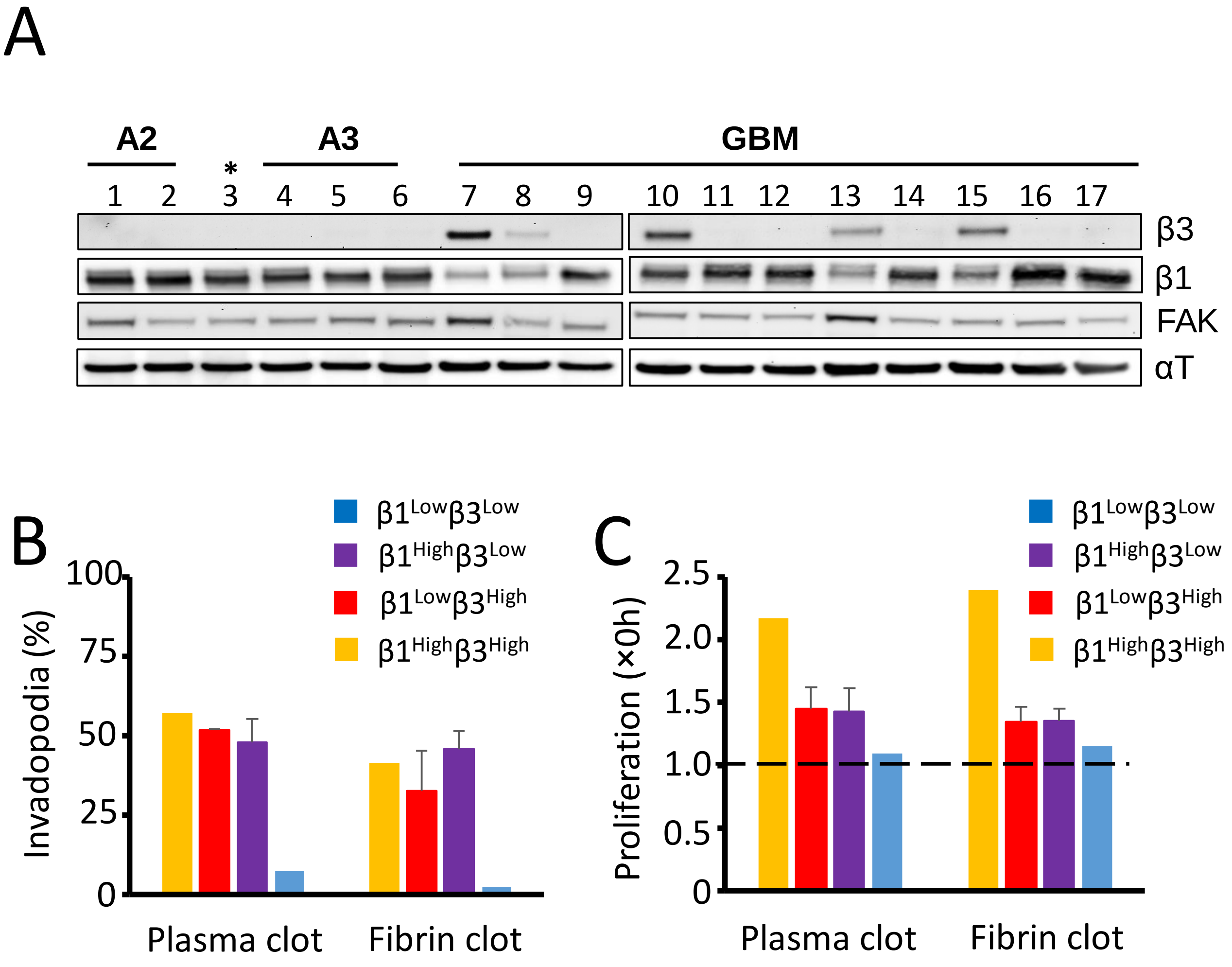

Knowles et al. Supplementary Fig. 5

Supplementary Fig. 5 *Expression of integrin β1 and integrin β3 affects invadopodia formation and growth of primary glioblastoma cells in fibrin clot and plasma clot.* (A), western blot analysis of integrin β1, β3 and FAK expression in a panel of primary glioma cells freshly isolated from patients with astrocytoma grade 2-4 (A2, A3 and GBM). α-tubulin (αT) served as a loading control. * refers to a patient originally classified as an astrocytoma Grade 2, but reclassified as a glioblastoma Grade 4. (B-C), primary GBM cells from patients (n = 12) were sorted for integrin β1 and integrin β3 protein expression based on western blot analysis to generate 4 subgroups: $\beta1^{Low}\beta3^{Low}$ (n = 1; patient 8), $\beta1^{High}\beta3^{Low}$ (n = 7; patients 3, 9, 11, 12, 14, 16 and 17), $\beta1^{Low}\beta3^{High}$ (n = 3; patients 7, 13 and 15) and $\beta1^{High}\beta3^{High}$ (n = 1, patient 10). Cells were embedded in a 3D matrix of plasma clot or fibrin clot and invadopodia-positive tumor cells were counted as percent of total per optical field after 4 days of embedding (B). Proliferation was assessed per optical field as fold increase of cells after 4 days of embedding (C). The dotted line reflects baseline cell numbers.
