# Supplementary material for: Clotting Promotes Glioma Growth and Infiltration Through Activation of Focal Adhesion Kinase
Source: Cancer Res Commun. 2024 Dec 13;4(12):3124–36. doi: 10.1158/2767-9764.CRC-24-0164 (PMC11638908; doi:10.1158/2767-9764.CRC-24-0164)
Supplement: Supplementary Fig. 6 — FAK activation in glioblastoma cells embedded in fibrin clot [file crc-24-0164_supplementary_fig.6_suppsf6.pdf]

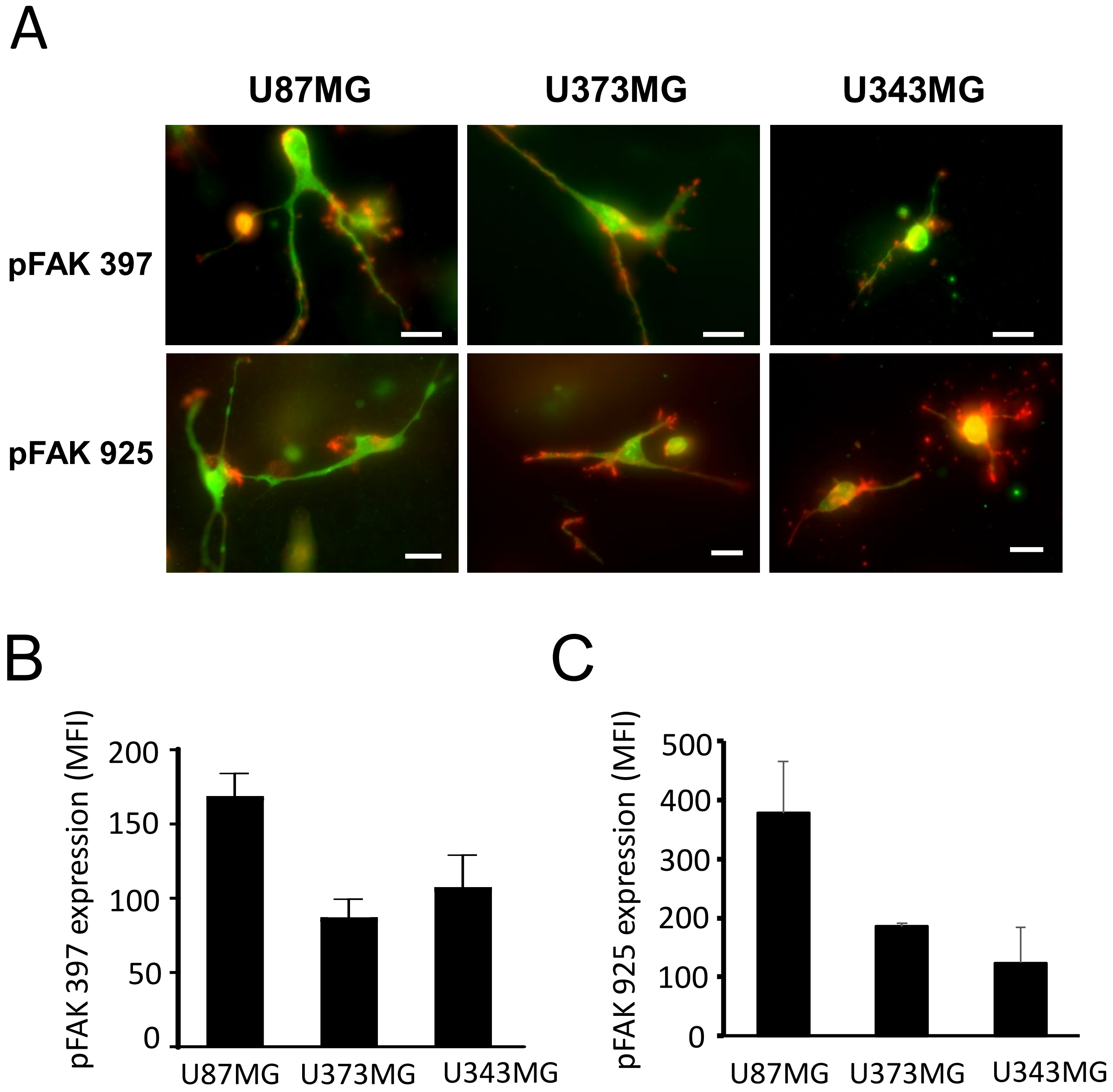

Knowles et al. Supplementary Fig. 6

Supplementary Fig. 6 *FAK activation in glioblastoma cells embedded in fibrin clot.* (A)*,* U87MG, U373MG and U343MG cells were fixed 2 days after embedding in fibrin, stained for anti-pFAK Y397 (Thermo Fisher Scientific, catalog # 700255; upper panel, green) and anti-pFAK Y925 (Cell Signaling, catalog # 3284; lower panel, green) and analyzed by fluorescence deconvolution microscopy. Actin is stained in red. Representative images are shown. Scale bar, 20 µm. (B-C), mean fluorescence intensity (MFI) of pFAK 397 (B) and pFAK 925 (C) expression measured using Zen 3.2 software (Zeiss). MFI represents pFAK expression minus signal intensity from nonspecific control IgG (R&D Systems, catalog # AB-105-C).
