# Supplementary material for: Clotting Promotes Glioma Growth and Infiltration Through Activation of Focal Adhesion Kinase
Source: Cancer Res Commun. 2024 Dec 13;4(12):3124–36. doi: 10.1158/2767-9764.CRC-24-0164 (PMC11638908; doi:10.1158/2767-9764.CRC-24-0164)
Supplement: Supplementary Fig. 7 — Invadopodia formation of GBM cells in fibrin clot and plasma clot depends on integrins β1 and β3 [file crc-24-0164_supplementary_fig.7_suppsf7.pdf]

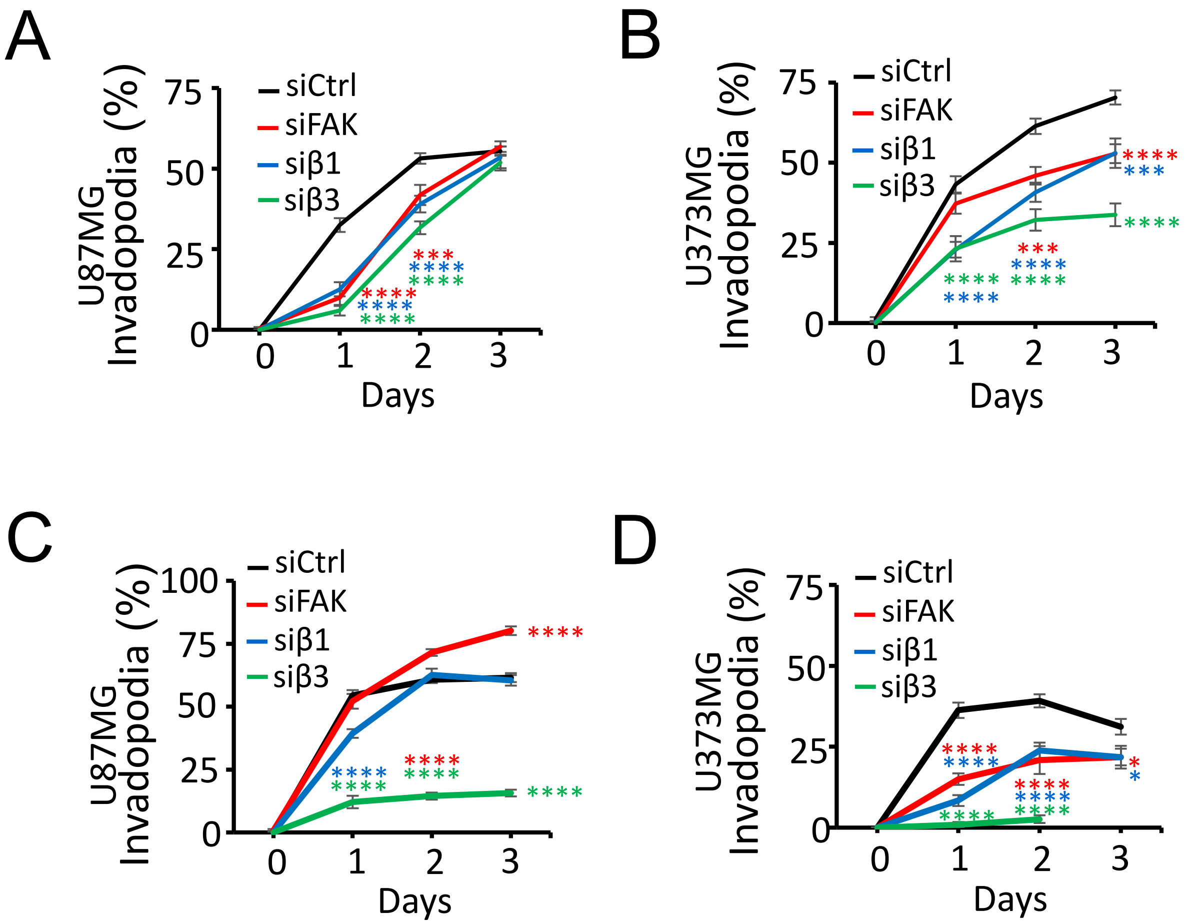

Knowles et al. Supplementary Fig. 7

Supplementary Fig. 7 *Invadopodia formation of GBM cells in fibrin clot and plasma clot depends on integrins β1 and β3.* (A-D), invadopodia formation was analyzed over time in plasma clot embedded U87MG (A) and U373MG (B) cells and in fibrin clot embedded U87MG (C) and U373MG (D) cells following transfection with siβ1, siβ3 and siFAK compared to siCtrl using phase contrast microscopy. No data is shown for U373MG transfected with siβ3 in fibrin after 48 hours as the fibrin clots lysed. *, $P<0.05$; **, $P < 0.01$; ***, $P<0.001$; ****, $P < 0.0001$ compared to siCtrl.
