# Supplementary material for: Clotting Promotes Glioma Growth and Infiltration Through Activation of Focal Adhesion Kinase
Source: Cancer Res Commun. 2024 Dec 13;4(12):3124–36. doi: 10.1158/2767-9764.CRC-24-0164 (PMC11638908; doi:10.1158/2767-9764.CRC-24-0164)
Supplement: Supplementary Fig. 8 — Expression of Integrin β1 and integrin β3 in gliomas based on IDH1 and EGFR mutation status [file crc-24-0164_supplementary_fig.8_suppsf8.pdf]

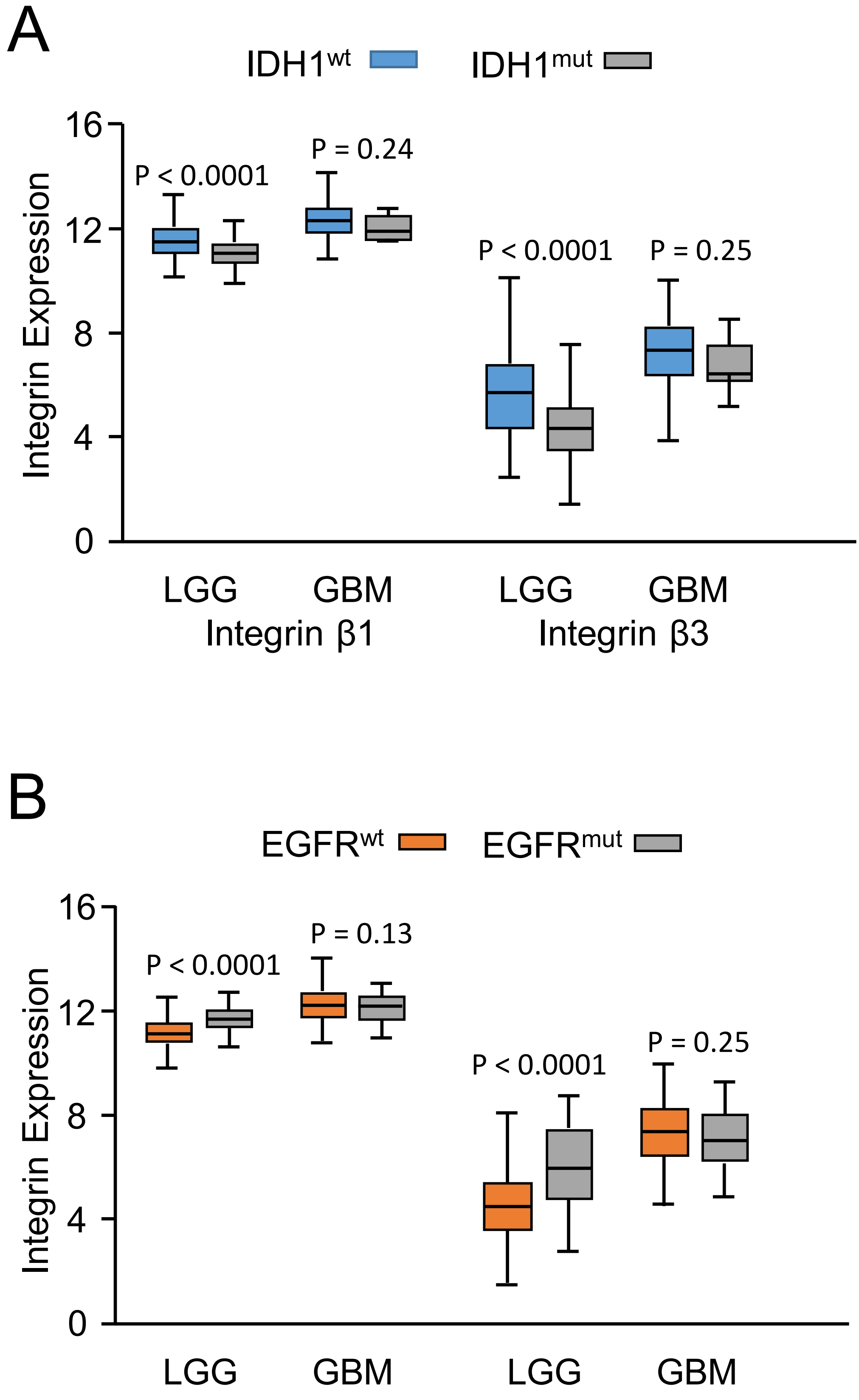

Knowles et al. Supplementary Fig. 8

Supplementary Fig. 8 *Expression of Integrin β1 and integrin β3 in gliomas based on IDH1 and EGFR mutation status.* (A), integrin β1 (left) and integrin β3 (right) mRNA levels in tumor tissue from patients with lower grade glioma (LGG, WHO grade 2 and 3, n = 507) or glioblastoma (GBM, n = 149) were obtained from The Cancer Genome Atlas (TCGA) and analyzed for missense/inframe mutations in isocitrate dehydrogenase (IDH1) using The UCSC Xena Functional Genomics Explorer software (LGG: $IDH1^{wt}$ n = 112, $IDH1^{mut}$ n = 395; GBM: $IDH1^{wt}$ n = 142, $IDH1^{mut}$ n = 7). Values are expressed as log2(norm_count+1) with the median (line), range (box) and standard deviation (bars) indicated for each group. (B), integrin β1 and integrin β3 mRNA levels sorted for EGFR mutation status (LGG: $EGFR^{wt}$ n = 471, $EGFR^{mut}$ n = 36); GBM: $EGFR^{wt}$ n = 105, $EGFR^{mut}$ n = 44). The number of EGFR mutations represents the sum of deleterious, missense/inframe, silent and intron mutations.
