# Supplementary material for: Clotting Promotes Glioma Growth and Infiltration Through Activation of Focal Adhesion Kinase
Source: Cancer Res Commun. 2024 Dec 13;4(12):3124–36. doi: 10.1158/2767-9764.CRC-24-0164 (PMC11638908; doi:10.1158/2767-9764.CRC-24-0164)
Supplement: Supplementary Video Legend — Legend for supplementary videos 1-6 [file crc-24-0164_supplementary_video_legend_suppsv1-6.docx]

**SUPPLEMENTARY VIDEO LEGENDS**

**Supplementary Videos:** U87MG were embedded in fibrin clot and monitored by real-time video microscopy over 3 days using the ImageXpress Micro XLS Widefield High Content Screening System (Molecular Devices). U373MG and U343MG are shown in fibrin clot during day 1 and U87MG cells are shown in 3D matrigel basement membrane (Corning). Supplemental Video 1: U87MG in fibrin clot day 1; Supplemental Video 2: U87MG in fibrin clot day 2; Supplemental Video 3: U87MG in fibrin clot day 3; Supplemental Video 4: U373MG in fibrin clot day 1; Supplemental Video 5: U343MG in fibrin clot day 1; Supplemental Video 6: U87MG in matrigel day 1.
