# Supplementary material for: Clotting Promotes Glioma Growth and Infiltration Through Activation of Focal Adhesion Kinase
Source: Cancer Res Commun. 2024 Dec 13;4(12):3124–36. doi: 10.1158/2767-9764.CRC-24-0164 (PMC11638908; doi:10.1158/2767-9764.CRC-24-0164)
Supplement: Supplementary Table 1 — Clinicopathologic data of tissue donors [file crc-24-0164_supplementary_table_1_suppst1.docx]

**Supplementary Table 1** Clinicopathologic data of tissue donors.
